# Cross‐species rescue reveals sequence requirements for a rapidly evolving intrinsically disordered region

**DOI:** 10.1371/journal.pbio.3003396

**Published:** 2025-09-25

**Authors:** Sang On Park, Rishad Khondker, Ariel Blank, Annie Dyatel, Corey Frazer, Richard J. Bennett, Robert J. D. Reid, Rodney Rothstein, Luke E. Berchowitz

**Affiliations:** 1 Department of Genetics and Development, Hammer Health Sciences Center, Columbia University Irving Medical Center, New York, New York, United States of America; 2 Department of Molecular Microbiology and Immunology, Brown University, Providence, Rhode Island, United States of America; 3 Taub Institute for Research on Alzheimer’s and the Aging Brain, New York, New York, United States of America; Stowers Institute for Medical Research, UNITED STATES OF AMERICA

## Abstract

Many proteins contain intrinsically disordered regions (IDRs) that are essential for their function but do not adopt a stable structure; instead, they exist as an ensemble of conformations. Because these regions lack fixed structural constraints, traditional structure-based and alignment-based approaches are often ineffective for studying their sequence–function relationships. Here, we present an approach that combines molecular evolution with genetic complementation to extract functional sequence features of an IDR. We use the budding yeast RNA-binding protein Rim4 as a model system. Rim4 is required for sporulation, and its IDR facilitates its dual role as a translational activator and repressor. Notably, Rim4’s IDR supports assembly into an SDS-resistant amyloid-like form, which is required for its repressor function. We demonstrate that the Rim4 IDR is functionally conserved across orthologous sequences spanning more than 400 million years of evolution, despite extensive sequence divergence. Our results suggest that noncomplementing Rim4 IDRs generally evolve toward higher hydrophobicity, and that reducing hydrophobicity can refunctionalize a nonfunctional IDR sequence that diverged over 200 million years ago. In the refunctionalized IDR, the activator function is restored, whereas assembly into an amyloid-like form remains uncomplemented. Overall, our findings add to evidence that IDRs can perform multiple functions, with each role optimized by distinct biochemical properties, and that evolutionary pressure favoring one function may drive the IDR toward biochemical characteristics that compromise its other functions.

## Abstract 2

Intrinsically disordered regions (IDRs) in proteins can perform multiple functions despite lacking stable structure and conserved sequence. This study uses cross-species rescue of yeast Rim4 to reveal how evolutionary pressure on IDRs can favor biochemical traits underlying distinct functions, influencing functional conservation and refunctionalization across hundreds of millions of years.

## Introduction

Despite a lack of a stable three-dimensional structure, intrinsically disordered regions (IDRs), which are widespread within proteomes [1], are essential for numerous important protein functions [2]. Disordered chains of amino acids can function simply as flexible linkers, enabling spatial orientation of folded domains, or as entropic bristles that enhance protein solubility by creating a more favorable environment for water interactions [3,4]. The inherent flexibility and accessibility of IDRs also facilitate post-translational modifications, which can profoundly influence their biochemical properties and regulation [5–8]. Additionally, IDRs mediate interactions with other proteins, nucleic acids, and lipids [9,10], positioning them as key hubs of signaling networks [11,12]. More recently, IDRs have been studied extensively in the context of biomolecular assemblies where they play a pivotal role in the formation of nonmembrane-bound compartments that sequester or concentrate cellular components [13–16]. IDR-mediated assembly often relies on multivalency, providing a flexible and accessible platform for distributing short interaction modules [17]. Although IDRs often diverge rapidly at the sequence level, recent work has begun to connect specific biophysical features—such as patterned charge distributions, aromatic and hydrophobic residue content, and low‐complexity motifs—to distinct regulatory activities [18–21]. Nonetheless, a comprehensive understanding of how these and other sequence properties combine to specify individual IDR functions is far from complete.

Protein function is typically understood via a structure and sequence alignment-based paradigm, where function is deduced through structural analysis or comparisons of conserved sequences and structures. IDRs, however, exist as dynamic ensembles of configurations and, by definition, do not adopt stable structures under physiological conditions. As a result, these regions cannot be studied using traditional structure-based frameworks. Standard sequence alignment-based analyses are also limited in their utility: without rigid structural constraints, IDRs evolve rapidly, leading to widespread sequence variations that complicate alignments [22]. Despite the rapid evolution of sequences, certain features within IDRs remain conserved, and specific patterns within IDRs can predict their functions [23,24]. Although orthologous IDRs are typically conserved with regard to disorder, their amino acid sequences vary, underscoring that while disorder is often an important functional constraint, the sequences that support it are flexible [25].

Because of the challenges inherent to uncovering sequence–function relationships in IDRs, researchers have developed alternative methods to analyze these regions. For example, given that IDR conformation correlates with function and localization, one can determine which sequence features best distinguish an IDR’s conformational properties [26]. Another approach is to systematically extract conserved sequence features from a group of heterologous IDRs, which can be used to model feature–function relationships [23,24,27]. We reasoned that a phylogenetically informed molecular evolution approach based on genetic complementation of an IDR would allow us to extract functional features enriched in complementing compared to noncomplementing sequences [28].

To facilitate our approach, we required a protein with the following properties: 1) The protein must harbor an IDR which is clearly required for a known function, 2) disruption of the IDR results in a nonlethal phenotype that is robust, quantifiable, and rescuable, and 3) the gene encoding the protein must be conserved and identifiable within a broad range of genomes. Rim4 is a yeast RNA-binding protein (RBP) that fits these criteria. Early in sporulation, its expression peaks and it activates premeiotic DNA replication and chromosome segregation; *rim4Δ* cells arrest before meiotic entry and fail to form spores [29,30]. As meiosis progresses, Rim4 adopts a second role as a translational repressor, selectively blocking translation of target mRNAs, including *Ty3* retrotransposon transcripts and genes required for meiosis II and spore formation [31,32]. Rim4 harbors three RNA recognition motifs (RRMs) and a C-terminal IDR, which contains a prion-like domain (PrLD) designated by a polyasparagine (polyN) tract [33]. What is unusual about Rim4 is that its function as a translational repressor is activated by formation into an SDS-resistant amyloid-like assembly, which is supported by its C-terminal IDR [34]. We describe Rim4 structures “amyloid-like” based on their SDS-resistance, without implying a link to pathological amyloids. Rim4 binds to and represses its RNA targets orders of magnitude more efficiently in its amyloid-like assembled state compared to its monomeric state, revealing a causal connection between assembly and function [35].

Observations raise the possibility that Rim4’s IDR plays roles in both activation and repression. Rim4 was first identified by the dramatic loss of Ime2 protein in *rim4Δ* cells [36], even though *IME2* mRNA remains abundant in *rim4Δ* and IDR-truncated strains (S1A Fig), revealing a post-transcriptional activation defect, though we cannot exclude that this arises via repression of a negative regulator. Further supporting an activator role, *RIM4* overexpression alleviates the meiotic-entry defects of *kar4Δ* mutants, which are deficient in a cofactor necessary for m6A RNA methylation, a process uniquely required for meiotic entry in yeast [37]. Whether this rescue depends on Rim4’s IDR remains unknown. Alongside its later, well-established role in repressing translation of select mRNAs, these findings suggest a dual-mode model similar to those of DAZL [38] and Orb2 [39], in which Rim4 can both activate and repress translation. Lastly, the severe prophase-entry defect of **rim4*Δ*IDR** mutants is consistent with the idea that the IDR could contribute both to early meiotic activation and to later repressive functions.

Using Rim4 as a model, we employed genetic complementation and evolutionary analysis to dissect the sequence–function relationships of an IDR. Disorder alone proved insufficient to fulfill function; specific sequence features were required. Despite this specificity, a wide range of sequence space was able to support Rim4 function. Complementing IDRs maintained a characteristic range of hydrophobicity compared to noncomplementing IDRs, and altering hydrophobicity could both abrogate the function of the *Saccharomyces cerevisiae* Rim4 IDR and restore activity to a decayed noncomplementing IDR. More broadly, our results illuminate the balance between functionality and pathological aggregation in RBPs, which are enriched in IDRs and form pathological aggregates in several severe human diseases [40].

## Results

### The Rim4 IDR contains specific functional determinants

IDRs of assembly-forming proteins are sometimes modular and can be substituted freely from species as diverged as yeast and humans [13,33,41]. IDRs, particularly those harboring a PrLD, can drive assembly formation, which can account for some, if not all, of their function [42,43]. Indeed, numerous examples exist where heterologous IDRs can promote assembly and do [44,45] or do not [46] rescue protein function (reviewed in [28]). If the Rim4 C-terminal IDR supports function simply by being disordered, Rim4 should be able to function even when this region is replaced with the IDR of another protein with similar molecular properties. By identifying sequence features shared among sequences that complement (and depleted in those that do not), we reasoned that we could gain insight into the molecular requirements of IDRs in RBPs that function in translational control. This analysis is aided by the fact that the Rim4 C-terminal IDR is essential for meiosis and sporulation, and meiotic completion and kinetics can be used as quantitative readouts for function.

**Fig 1:**
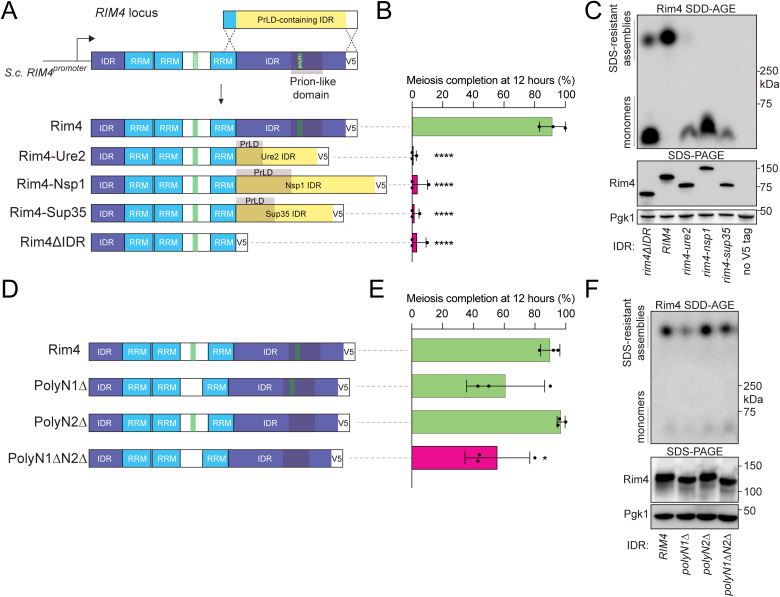
Heterologous yeast prion-like domain-(PrLD) containing intrinsically disordered regions (IDRs) do not complement the Rim4 IDR. **(A)** Schematic of PrLD-containing IDR substitutions and diagrams of Rim4-PrLD-containing IDR chimeras. RNA recognition motif (RRM) domains are colored in blue, polyN tracts in green, and IDRs in purple (Rim4) or yellow (heterologous). **(B, C)** Diploid strains homozygous for V5-tagged PrLD-containing IDR chimeras were induced to sporulate at 30°C. Meiotic progression was monitored by DAPI staining of nuclei. (B) Tetranucleate percentage (*i.e.*, completion of meiosis) at 12 h is shown, and detailed progression data is provided in S1C Fig. Each bar represents the mean of 3 biological replicates and error bars indicate SEM. Statistical significance was calculated using an ordinary one-way ANOVA where tetranucleate counts for each strain were compared to tetranucleate counts of wild type. Green bars indicate strains statistically indistinguishable from wild type (complementing), and magenta bars indicate strains with a significant sporulation defect (noncomplementing). (C) Presence of Rim4 SDS-resistant assemblies at 6 h was tested using semi-denaturing detergent agarose gel electrophoresis (SDD-AGE). Pre-assay Rim4 protein levels were assessed by sodium dodecyl sulfate-polyacrylamide gel electrophoresis (SDS-PAGE) and immunoblot with Pgk1 as a loading control. **(D)** Diagrams of Rim4 polyN deletion mutants. **(E, F)** Diploid strains homozygous for polyN deletion alleles were induced to sporulate at 30°C. (E) Meiotic progression was analyzed by DAPI staining. (F) Presence of Rim4 SDS-resistant assemblies was determined by SDD-AGE, and total Rim4 levels were determined by SDS-PAGE immunoblot. * = *P* value ≤ 0.0332; ** = *P* value ≤ 0.0021; *** = *P* value ≤ 0.0002; **** = *P* value < 0.0001. The data underlying this figure can be found in S1 Data.

To test whether the Rim4 IDR operates under molecular constraints similar to other PrLD-containing IDRs, we asked whether IDRs from other yeast proteins could rescue Rim4ΔIDR function. Leaving the Rim4 RRMs and N-terminal IDR intact, we replaced the Rim4 C-terminal IDR with IDRs from three budding yeast proteins (Sup35, Ure2, and Nsp1, Fig 1A). The N and Q-rich PrLDs of Sup35 and Ure2 and FG repeats of Nsp1 all form self-seeded amyloids in vitro but often require the prion form of the *[PIN+]* seeding factor Rnq1 to form in vivo [47–51]. Although our SK1 strain was *[pin-]* (S1B Fig), nonamyloid Sup35 assemblies are still possible in a *[pin-]* background [52], suggesting that substituted IDRs may still rescue Rim4 function. We compared meiotic kinetics in these IDR replacement strains to wild type and **rim4*Δ*IDR** (a truncation lacking the C-terminal 271 residues). All three IDR-substitution mutants failed to initiate meiosis by hour 12 in sporulation medium and were indistinguishable from **rim4*Δ*IDR** mutants (Figs 1B and S1C). We then tested whether the Sup35, Ure2, or Nsp1 IDRs were sufficient to drive assembly into an amyloid-like assembly using semi-denaturing detergent agarose gel electrophoresis (SDD-AGE) [53]. While wild type Rim4 readily assembles into an SDS-resistant form, none of the substitution proteins formed assemblies (Fig 1C). We note that Rim4ΔIDR still produces the same large SDD-AGE species as wild type, albeit at reduced levels, indicating that the IDR supports but is not essential for assembly, in contrast to our prior conclusions. The IDR is unlikely to constitute part of the SDS-resistant core. Instead, it may promote oligomerization or phase separation that precedes the SDS-resistant state, or interact with chaperones that regulate assembly and disassembly, as observed for Sup35 amyloids [54]. Our data demonstrate that other PrLD-containing IDRs substitute neither assembly nor function of the Rim4 IDR. While these results do not inform whether the Rim4 IDR functions by driving assembly, they demonstrate that the Rim4 IDR does not function simply by being disordered and instead has distinct molecular requirements.

Because heterologous PrLD-containing IDRs failed to complement the Rim4 IDR, we asked whether the polyN tracts that define the Rim4 PrLD are necessary for function. We assessed Rim4 function in strains lacking the polyN region between RRM1 and RRM2 (*polyN1Δ*), the polyN region within the C-terminal IDR (*polyN2Δ*), and both (*polyN1ΔN2Δ*) (Fig 1D). Unexpectedly to us, neither single deletion mutant showed a significant effect on meiotic progression, while *polyN1ΔN2Δ* exhibited a modest defect (Fig 1E). Both *polyN1Δ* and *polyN1ΔN2Δ* showed moderate meiotic progression delays while *polyN2Δ* was essentially wild type (S1C Fig) indicating that the defect in *polyN1ΔN2Δ* is not driven by the polyN tract within the C-terminal IDR. All three mutant strains formed SDS-resistant assemblies similar to wild type (Fig 1F). Although the polyN tract within the Rim4 C-terminal IDR is a conspicuous feature, our results indicated that it plays a minor (if any) role in directing Rim4 assembly or function, and other features must be responsible.

### The Rim4 IDR is rapidly evolving while retaining function

**Fig 2:**
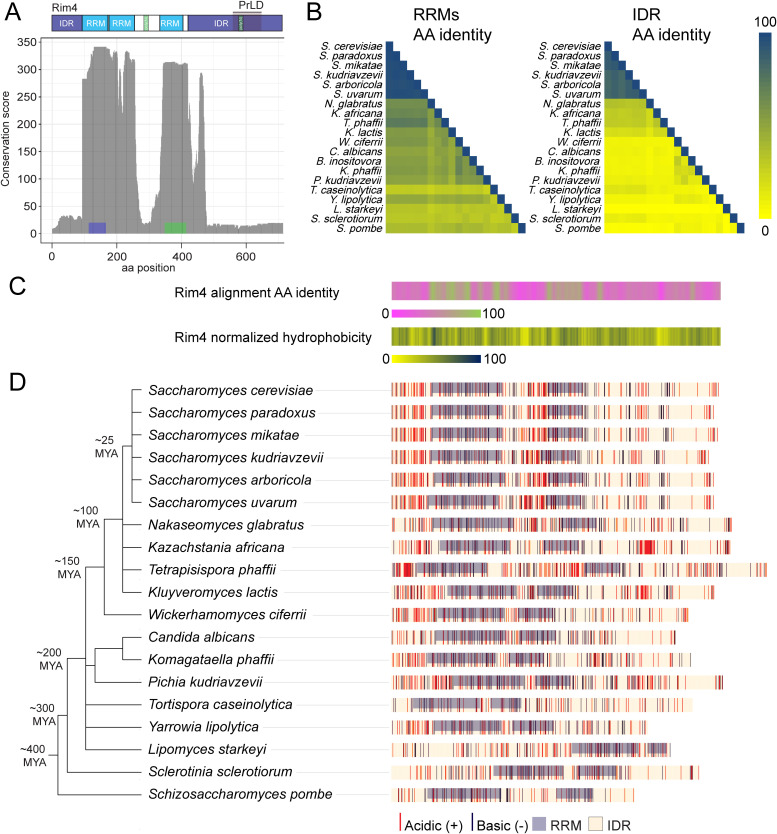
The sequences and features of the Rim4 intrinsically disordered region (IDR) are rapidly evolving. **(A)** 332 yeast genome sequences (spanning ~400 million years of evolution, from Shen 2018 [55]) were queried against *S*accharomyces* cerevisiae RIM4*. BLAST hits were grouped and filtered so that only one locus is represented by each genome. The y-axis indicates the number of the 332 genomes that generated a positive hit by BLAST at regions indicated on the x-axis. **(B)** For each pair of *RIM4* orthologs (verified by synteny analysis, S2 Fig), percentage of identical pairwise amino acid alignment was plotted for RNA recognition motifs (RRMs) (left) and IDR alignments (right). **(C)** Nineteen orthologous *RIM4* sequences were aligned using COBALT [56]. The top plot shows percentage sequence identity (green is 100% and magenta is 0%), and the bottom plot shows normalized hydrophobicity of Rim4 proteins using the Kyte–Doolittle hydropathy scale [57]. **(D)** Distribution of charged residues and RRM domains is plotted next to a phylogenetic tree of analyzed yeast species. The phylogenetic tree was built using PhytoT based on NCBI phylogenetic data and was visualized using interactive tree of life (iTOL) software [58]. RRM domains are colored light blue. Acidic residues (D, E) are colored in red and basic residues (R, K) in blue. The data underlying this figure can be found in S1 Data.

Similar to many IDR-containing proteins, the Rim4 C-terminal IDR is evolving rapidly compared to its RNA-binding domains. In multiple sequence alignments and BLAST searches, the structured RRM domains showed good conservation within *Saccharomyces sensu stricto*. On the other hand, *RIM4* BLAST searches of 332 budding yeast isolates [55] returned negligible hits for much of the IDR beyond *Saccharomyces* (Fig 2A). We hypothesized that functional features and properties are likely conserved among distantly related yeasts despite extensive sequence divergence. By assessing the degree to which *RIM4* orthologs could complement function in *S. cerevisiae*, we could categorize sequences into those that support function and those that do not.

Our first step was to identify and analyze the sequences of *RIM4* orthologs from other yeasts sampling broadly from the yeast phylogeny from *S. cerevisiae* to *Schizosaccharomyces pombe* (diverged ~400 million years ago [MYA]) [55]. BLAST searches with *S. cerevisiae* Rim4 protein sequence returned many potential orthologs, but these sequences yielded good alignment only on structured RRM domains. To determine genes that were bona fide *RIM4* orthologs, we combined our BLAST searches with a comparison of neighboring genes of orthologous *RIM4* loci (i.e., synteny analysis, S2 Fig). Analyzing syntenic blocks from *S. cerevisiae* to *S. pombe* revealed a significant overlap of neighboring genes among suspected *RIM4* loci. Conservation of these syntenic blocks supports the assertion that these loci encode *RIM4* orthologs. Given the essentiality of Rim4’s IDR for protein function, we aligned *RIM4* and its orthologs to assess sequence conservation. The majority of *RIM4* orthologs are predicted to encode long IDRs, and as expected, amino acid identity sharply drops beyond 25 MYA due to rapid evolution and changes in RRM orientation during evolution (Fig 2B and 2C).

To explore evolutionary conservation among *RIM4* orthologs, we analyzed the biochemical characteristics of the encoded proteins. For this analysis, we considered sequences C-terminal to the last RRM as IDRs with a few exceptions (S3A Fig). The *Lipomyces starkeyi* N-terminal sequence was used as an IDR given that its RRMs are positioned at the C-terminus. *Sclerotinia sclerotiorum* has IDRs of similar lengths on both N and C-termini, and the longer N-terminal sequence was chosen. We looked at distribution of hydrophobicity, charge, and aromatic residues whose patterning can determine IDR conformation [59–61]. The *S. cerevisiae* Rim4 has a distinct cluster of acidic residues in the middle of its C-terminal IDR, but this feature is absent outside of the *Saccharomyces* genus, either through intermixing with basic residues or separation of acidic residues (Fig 2D). Similarly, apparent clustering of aromatic residues deteriorated outside of *Saccharomyces* (S3B Fig). Clustering of hydrophobicity was better conserved and observed in the C-termini of most IDRs except for *Tortispora* **caseinolytica, L. starkeyi, S. sclerotiorum,** and *S. pombe*. Overall, the clustering of hydrophobic residues in the Rim4 IDRs across orthologs suggests that this pattern may be essential for function.

**Fig 3:**
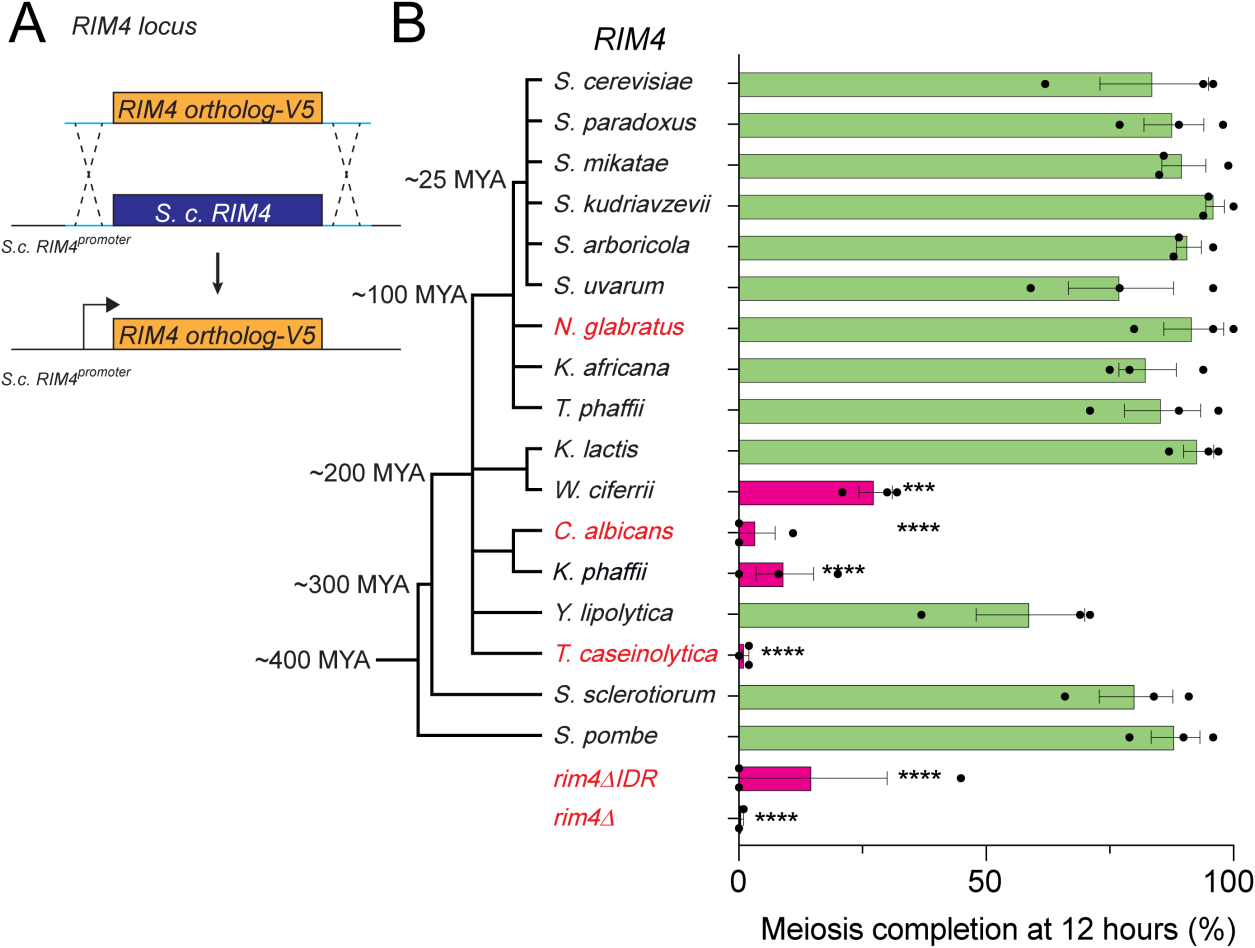
Rim4 function is conserved despite evolutionary sequence divergence. **(A)** Schematic showing markerless orthologous *RIM4* substitution. **(B)** *RIM4* orthologs and the phylogenetic relationships among the parent yeasts are indicated. Species that do not sporulate are written in red. Diploid strains homozygous for orthologous *RIM4* CDSs were induced to sporulate at 30°C. Meiotic progression was monitored by DAPI staining. (B) Tetranucleate percentage at 12 h is shown, and detailed progression data is provided in S4A Fig. Each bar represents 3 biological replicates and error bars indicate SEM. Statistical significance was calculated using an ordinary one-way ANOVA, where tetranucleate counts for each strain were compared to tetranucleate counts of wild type. * = *P* value ≤ 0.0332; ** = *P* value ≤ 0.0021; *** = *P* value ≤ 0.0002; **** = *P* value < 0.0001. The data underlying this figure can be found in S1 Data.

To analyze whether orthologous *RIM4* coding sequences (CDS) could support function in *S. cerevisiae*, we analyzed sporulation in 16 strains where the entire *RIM4* CDS is substituted with an orthologous *RIM4* gene (Fig 3A)*.* Most yeast species we analyzed sporulate except for *Nakaseomyces glabratus* [62], *Candida *albicans** [63], and *T. caseinolytica* [64]. Given that heterologous PrLD-containing IDRs from *S. cerevisiae* failed to support Rim4 function, we were surprised that most orthologous *RIM4* substitutions restored efficient meiotic progression and sporulation, as evidenced by the percentage of tetranucleate cells at 12 h in sporulation medium (Figs 3B and S4A). Only four orthologs (*Wickerhamomyces *ciferrii** [150–200 MYA]*, *C. albicans** [~200 MYA]*, *Komagataella phaffii** [~200 MYA], and *T. caseinolytica* [300–400 MYA]) failed to complement and instead resembled **rim4*Δ*IDR**. Interestingly, orthologs more evolutionarily distant (*S. sclerotiorum* and *S. pombe*) complemented despite having a shorter IDR or a different positioning architecture compared to that of the *S. cerevisiae* Rim4. *RIM4* orthologs from lineages that have lost sporulation were statistically less likely to complement function, consistent with relaxed evolutionary constraint on *RIM4* in those species (S4B Fig). Together, these results demonstrate that despite substantial divergence in IDR amino acid sequences, Rim4 function remains remarkably conserved, indicating that a broad range of sequences, provided they contain specific key features, can support its activity.

### Complementation by orthologs is driven by IDR sequence

**Fig 4:**
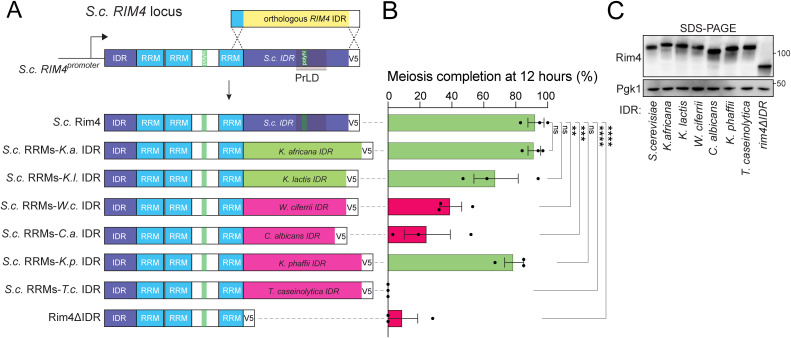
Complementation by *RIM4* orthologs is driven by their intrinsically disordered regions (IDRs). **(A)** Schematic of orthologous *RIM4* IDR substitution and diagrams of *Saccharomyces cerevisiae* RNA recognition motifs (RRMs)-ortholog IDR chimeras. Orthologous IDRs from complementing orthologs are colored in green and noncomplementing orthologs in magenta. **(B, C)** Homozygous IDR substitution strains were induced to sporulate at 30°C. Meiotic progression was monitored by DAPI staining of nuclei. (B) Tetranucleate percentage at 12 h is shown, and detailed progression data is provided in S5A Fig. Each bar represents 3 biological replicates and error bars indicate SEM. Statistical significance was calculated using an ordinary one-way ANOVA, where tetranucleate counts for each strain were compared to tetranucleate counts of wild type. (C) Rim4 protein levels in each strain at 6 h into meiosis were assessed by sodium dodecyl sulfate-polyacrylamide gel electrophoresis (SDS-PAGE) and immunoblot with Pgk1 as a loading control. * = *P* value ≤ 0.0332; ** = *P* value ≤ 0.0021; *** = *P* value ≤ 0.0002; **** = *P* value < 0.0001. The data underlying this figure can be found in S1 Data.

Because Rim4 can be broadly divided into ordered regions (RRM domains) and IDRs, we asked whether orthologous IDRs themselves were sufficient to support function in the context of *S. cerevisiae* RRMs. To test this idea, we assessed meiotic progression in strains expressing chimeric Rim4 proteins with *S. cerevisiae* Rim4 RRMs fused to orthologous IDRs (Fig 4A). We generated constructs using IDRs from two distant, but complementing orthologs (*Kazachstania africana* and *Kluyveromyces *lactis**) and all four noncomplementing orthologs (**W. ciferri*, *C. albicans*, *K. phaffii*,* and *T. caseinolytica*). Similar to the complete CDS replacements, chimeric constructs containing IDRs from complementing orthologs supported efficient meiotic progression, whereas those with noncomplementing IDRs exhibited reduced efficiency, with one notable exception (Figs 4B, 4C, and S5A). We found that the *K. phaffii* IDR fusion now showed complementation, indicating that this IDR is functional, and the noncomplementation we observed in the **K. phaffii* RIM4* whole gene substitution was likely driven by incompatible RRM domains.

**Fig 5:**
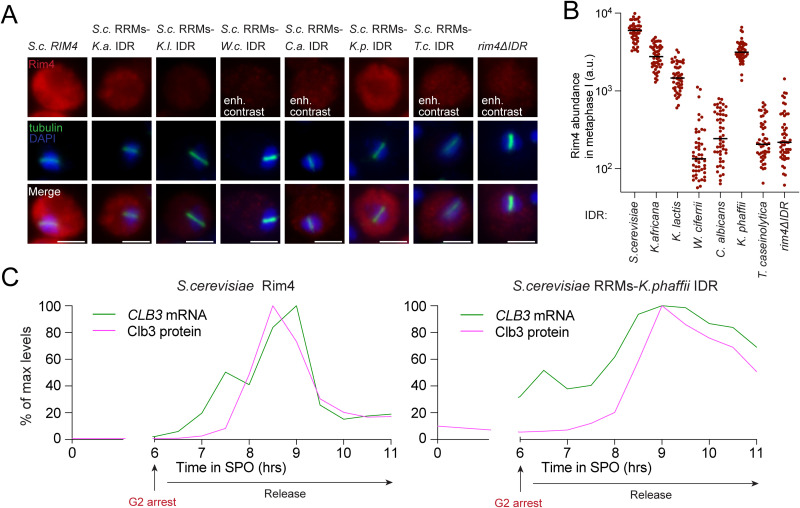
Complementing chimeric proteins are abundant and retain translational repression. **(A–C)** Diploid strains homozygous for *RIM4* intrinsically disordered region (IDR) substitutions and carrying the *NDT80-IN*, *GAL4.ER*, and *CLB3-3HA* alleles were induced to sporulate at 30°C. Six hours after induction, when cells had arrested in G2 due to the lack of *NDT80*, cultures were released by addition of 1 µM β-estradiol. (A) Representative immunofluorescence images of metaphase I cells stained for Rim4 (V5 epitope; red), α-tubulin (green), and DNA (DAPI; blue). Scale bar, 5 µm. Note that we enhanced the contrast in some strains to show Rim4 signal morphology. (B) Single-cell measurements for Rim4 levels in metaphase I were determined using V5 IF. Shown are individual measurements for 50 cells in each strain. (C) Clb3 protein and *CLB3* mRNA levels are plotted on the y-axis, and time in meiosis is plotted on the x-axis. Immunoblot and northern blot source data is shown in S6C Fig. The data underlying this figure can be found in S1 Data.

We next examined Rim4 IDR substitution constructs in sporulating cells by immunofluorescence and live imaging of C-terminal EGFP fusions (Figs 5A and S5B). All variants remained diffusely cytoplasmic, showing only intensity fluctuations rather than discrete foci or filaments. The exceptionally high abundance of Rim4 during meiosis likely masks any discrete assemblies, making morphological differences difficult to discern. By metaphase I, complementing constructs reached higher steady-state levels than noncomplementing ones (Fig 5B), suggesting that nonfunctional Rim4 proteins are unstable, selectively degraded, or inefficiently translated. To probe this, we measured protein stability by cycloheximide chase for each IDR fusion (S5C Fig). Although wild type Rim4 exhibited the slowest turnover, there was no clear correlation between stability and complementation. Together, these data support a model in which divergent noncomplementing IDR sequences in distant *RIM4* orthologs impair function in *S. cerevisiae*, possibly by altering translation efficiency.

Rim4 performs at least two functions during meiosis, and both require its IDR: it acts as a positive regulator of early meiotic genes [29,37] and as a translational repressor of transcripts that encode factors important for later meiotic events and spore formation [31]. At the onset of meiosis II, the Rim4 IDR is hyperphosphorylated, which triggers Rim4 disassembly and degradation to allow translation of previously repressed mRNA [7]. The first role likely underpins the phenotype of *rim4Δ* mutants; cells completely lacking *RIM4* function are defective in entry into pre-meiotic S-phase. While meiotic completion and kinetics cannot resolve early versus late functions of Rim4, early activity can be assayed through meiotic recombination or Ime2 accumulation, whereas late activity can be assessed by monitoring translational repression of a target such as *CLB3* during the first meiotic division. Because some chimeric RRM-IDR constructs support meiotic entry in *S. cerevisiae,* we asked whether they a) are properly cleared at meiosis II onset and b) form functional translational repressors. To facilitate these inquiries, we assessed Rim4 clearance and translational control in cells undergoing synchronized meiotic divisions using the *NDT80* block-release system [65]. In this system, the transcription factor *NDT80* is driven by the *GAL1-10* promoter, which is controlled by an estradiol-regulatable Gal4-ER fusion. Cells are arrested in meiotic G2 until β-estradiol is added, which synchronously releases the cells into the meiotic divisions. As expected, all complementing constructs entered and completed meiotic divisions (to various degrees) while all noncomplementing constructs failed to complete meiosis (S6A Fig). Additionally, we found that all complementing Rim4 IDR fusion constructs were properly cleared in meiosis II with kinetics similar to wild type (S6B Fig). Lastly, we compared translational control in wild type and the most distant complementing fusion construct (*S. cerevisiae* RRMs–*K. phaffii* IDR). We found that translational repression was intact, as indicated by the restriction of Clb3 protein to meiosis II despite accumulation of *CLB3* mRNA throughout meiosis (Figs 5C and S6C). We concluded that distantly related Rim4 IDRs have retained function and regulation despite their amino acid divergence.

Because Rim4’s translational repressor function in *S. cerevisiae* requires assembly into an SDS-resistant state, and this state is supported by its IDR, we next tested whether IDR substitution constructs could assemble using SDD-AGE. All constructs formed some degree of SDS-resistant assemblies, with no clear correlation between assembly and function, except for the dysfunctional *T. caseinolytica* IDR, which produced heterogeneous smears more typical of true amyloids (S7A Fig). All Rim4 assemblies migrated well into the gel, indicating they are not trapped in the wells and they are sensitive to boiling, ruling out covalent cross-linking or post-translational modifications (S7B Fig). Together, these observations suggest that the lack of complementation by nonnative IDRs is unlikely to stem from a failure to form SDS-resistant assemblies.

To test whether heterotypic interactions or functional interference occur when different Rim4 orthologs are co-expressed, we examined meiotic progression and sporulation efficiency in heterozygous *S. cerevisiae* diploids carrying one copy of *S. cerevisiae RIM4* and one copy of an orthologous *RIM4* (S7C Fig). In all combinations, including those with nonfunctional orthologs, meiosis progressed efficiently and Rim4 assemblies were observed (S7D and S7E Fig). SDD-AGE could not differentiate whether cells contained separate homotypic assemblies or mixed heterotypic assemblies. Nonetheless, our results indicate that cells can tolerate expression of at least two Rim4 isoforms without generating dysfunctional molecular interactions.

### Complementing Rim4 IDRs are tuned for a specific hydrophobicity range

We next attempted to extract sequence features that differentiate complementing and noncomplementing IDRs and thus would be candidate parameters that influence IDR function. Complementing IDRs included the *Saccharomyces sensu stricto*, *K. africana, K. lactis*, and *K. phaffii*. Noncomplementing IDRs (verified by chimeric analysis) included **W. ciferrii*, *C. albicans*,* and *T. caseinolytica*. We first compared length, disorder scores (PLAAC [66], IUPred [67,68], Metapredict [69]), and hydrophobicity. To interrogate changes in charge and hydrophobicity patterning, which influence protein compaction, we analyzed sequence charge decoration (SCD [59]) and sequence hydropathy decoration (SHD [60]). Higher SHD values suggest clustering of hydrophobicity, and comparatively negative SCD values suggest clustering of charge. We also measured distribution of each AA compared to all other AAs using Gini coefficient. We analyzed PKA and 14-3-3 consensus motifs [70,71] because phosphorylation by PKA triggers Rim4 degradation [35] and 14-3-3 proteins facilitate Rim4 phosphorylation [72–74]. Lastly, we analyzed IDR conformation measured in radius of gyration or end-to-end distance [75]. These results are summarized in S1 Table.

Among these comparisons, the parameters that (statistically) differentiated complementing and noncomplementing IDRs were PLAAC score, hydrophobicity, IUPred score, numbers of PKA sites, end-to-end distance, and valine content. Complementing IDRs tended to have higher disorder scores, whereas noncomplementing IDRs were more hydrophobic, a property partly attributable to their elevated valine content. Complementing IDRs may have a more compact dimension of ensemble average, shown by a lower end-to-end distance. Noncomplementing IDRs contained fewer PKA sites and may have evolved an alternative degradation pathway. We hypothesized that, depending on a yeast’s ecological niche and sporulation needs, its *RIM4* IDR was gradually selected to be either more hydrophobic or more charged and hydrophilic. These changes are reflected in differences in disorder scores and amino acid composition, and they may affect Rim4’s activator and/or repressor functions. A caveat to these analyses is that we did not correct IDRs for evolutionary distance, and we had more closely related IDRs within the complementing group. For this and other reasons, it was important for us to experimentally test the functional relationships supported by our feature comparisons.

### Hydrophobicity is a determinant of Rim4 IDR function

**Fig 6:**
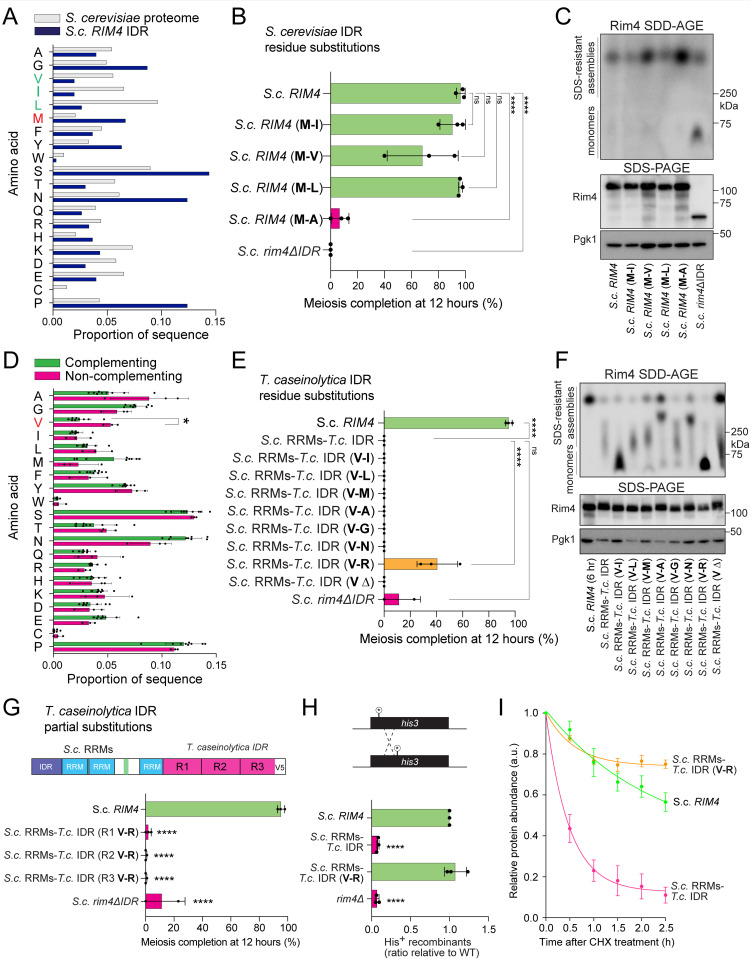
Rim4 intrinsically disordered region (IDR) function requires a specific hydrophobicity range. **(A)** The amino acid composition of the *Saccharomyces cerevisiae* Rim4 IDR was compared to the *S. cerevisiae* proteome. Nonaromatic hydrophobic amino acids valine, isoleucine, and leucine are highlighted in green, and methionine is highlighted in red. **(B, C)** Diploid strains homozygous for various *S. cerevisiae* Rim4 IDR methionine substitution mutants were induced to sporulate at 30°C. Meiotic progression was monitored by DAPI staining of nuclei. (B) Tetranucleate percentage at 12 h is shown, and detailed progression data is provided in S8A Fig. Each bar represents 3 biological replicates and error bars indicate SEM. Statistical significance was calculated using an ordinary one-way ANOVA, where tetranucleate counts for each strain were compared to tetranucleate counts of wild type. (C) Presence of Rim4 SDS-resistant assemblies at 6 h was tested using semi-denaturing detergent agarose gel electrophoresis (SDD-AGE). Pre-assay Rim4 protein levels were assessed by sodium dodecyl sulfate-polyacrylamide gel electrophoresis (SDS-PAGE) and immunoblot with Pgk1 as a loading control. **(D)** The amino acid composition of the complementing Rim4 IDRs was compared to noncomplementing IDRs. Statistical significance was calculated using unpaired *t* tests with Holm–Šídák multiple comparison correction. * = *P* value ≤ 0.05. **(E–G)** Diploids homozygous for various *Tortispora *caseinolytica** IDR valine substitution mutants (chimeric with *S. cerevisiae* RNA recognition motifs [RRMs]) were induced to sporulate at 30°C. (E) Meiotic progression was monitored by DAPI staining, and detailed progression data is provided in S8B Fig. (F) Presence of Rim4 SDS-resistant assemblies was determined by SDD-AGE, and total Rim4 levels were determined by SDS-PAGE/immunoblot. (G) Regions (sequential thirds) mutated V→R are shown in the diagram. **(H)** *HIS3* heteroallele recombination assay. Diploid strains carrying two *his3* point mutations in trans, and homozygous for one of four *RIM4* variants—wild type, *rim4Δ* (defective in prophase I entry), or *S. cerevisiae* RRMs*–T. caseinolytica* IDR fusion, with and without V→R substitutions, were induced to sporulate at 30°C for 24 h. Cultures were plated on histidine-dropout medium, and the frequency of His⁺ recombinants, indicating successful entry and progression through prophase I, is plotted relative to wild type. Each bar represents 3 biological replicates, and error bars indicate SEM. Statistical significance was calculated using an ordinary one-way ANOVA where recombination frequency for each strain was compared to wild type. **(I)** Cycloheximide chase analysis of Rim4 stability during meiosis. At 4 h after transfer to sporulation medium, cycloheximide was added, and samples were collected every 30 min for 2.5 h. Strains expressing wild type Rim4, the *S. cerevisiae* RRMs–*T. caseinolytica* IDR fusion, or that fusion bearing V→R substitutions were lysed, resolved by SDS-PAGE, and immunoblotted. Data from 3 biological replicates are normalized to the abundance at the time of cycloheximide addition (*t* = 0) and fitted with a one-phase exponential decay curve. * = *P* value ≤ 0.0332; ** = *P* value ≤ 0.0021; *** = *P* value ≤ 0.0002; **** = *P* value < 0.0001. The data underlying this figure can be found in S1 Data.

Because valine content, known to drive hydrophobicity and counteract disorder, appeared to be the primary factor distinguishing the two classes of IDRs, we sought to experimentally evaluate how hydrophobicity affects Rim4 IDR function. We chose to manipulate methionine residues because of their intermediate hydrophobicity and abundance within the Rim4 IDR (Fig 6A). *Saccharomyces* Rim4 IDRs are methionine-rich, which is similar to Pab1 and Pbp1, which are both yeast RBPs that form functional IDR-mediated assemblies [16,76]. We assessed Rim4 function in four *S. cerevisiae* Rim4 IDR variants where we replaced methionine residues with alanine (M→A, decreased hydrophobicity) or valine, isoleucine, or leucine (increased hydrophobicity). We found that replacing M→A resulted in a severe functional defect (Figs 6B and S8A). On the other hand, function was unaffected when we replaced methionine with isoleucine, leucine, or valine. All constructs readily assembled into SDS-resistant assemblies (Fig 6C). These results support the following conclusions: a) sufficient hydrophobicity is important for Rim4 IDR function, b) that methionine function in this context is not primarily based on its capacity to become modified, and c) assembly is insufficient to drive Rim4 function.

Given that noncomplementing orthologous IDRs are proportionally higher in valine (Fig 6D), we asked whether they could be refunctionalized by altering their hydrophobicity. For this purpose, we focused on the *T. caseinolytica* IDR, which harbors a decayed Rim4 IDR as evidenced by its noncomplementation phenotype, which resembles **rim4*Δ*IDR** and is the most distant noncomplementing ortholog we tested. The *T. caseinolytica* IDR sequence is rich in valine, and we substituted valine with amino acids that span the hydrophobicity scale from the most hydrophobic isoleucine to least hydrophobic arginine. Remarkably, we found that meiotic entry and progression could be partially rescued when we substituted valine with arginine (V→R, Figs 6E and S8B). Unexpectedly, we found that decreased hydrophobicity generally corresponded to increased formation of SDS-resistant assemblies (Fig 6F). V→R, however, did not rescue assembly, marking our first case where we were able to observe (partial) function in the absence of SDS-resistant assemblies. The rescue observed with the V→R substitution may reflect not only decreased hydrophobicity but also arginine-specific features—such as its positive charge or methylation potential—since the V→N mutant, which lowers hydrophobicity, albeit to a lesser degree, failed to complement. To test whether restored Rim4 function in the *T. caseinolytica* V→R IDR variant reflects a global reduction in hydrophobicity (or another arginine-related property) rather than the reintroduction of a specific regional motif, we measured meiotic function in chimeras in which we replaced valine with arginine in each successive third of the *T. caseinolytica* IDR (Figs 6G and S9). None restored Rim4 function, indicating that rescue likely depends on bulk effects rather than any discrete motif or local effect.

We hypothesized that excessive IDR hydrophobicity inhibits Rim4’s early activator function, raising the possibility that the V→R substitutions could restore this activity. To assess the extent of restoration, we measured intragenic meiotic recombination at *HIS3*, an early meiotic event that initiates during pre-meiotic S-phase and must be completed before the meiotic divisions [77,78]. After 24 h of sporulation, the V→R construct restored recombination to wild type levels, whereas the original *T. caseinolytica* IDR remained nonfunctional, similar to *rim4Δ* (Fig 6H). To determine whether altered protein turnover underlies the functional rescue by the V→R substitution, we next compared meiotic protein stability of the *T. caseinolytica* IDR and its V→R variant. The V→R variant exhibited dramatically increased stability relative to the native sequence (Fig 6I). Together, these results support a model in which Rim4’s early activator function requires IDR hydrophobicity to lie within a functional window—lowering hydrophobicity rescues activity by enhancing protein stability rather than by reconstituting SDS-resistant assemblies.

## Discussion

Genetic complementation studies, where heterologous IDRs restore function to mutant proteins, have been instrumental in linking specific IDR sequences and features to biological functions [19,21,28,42–44]. However, pinpointing the precise determinants of IDR function has remained difficult, in part because most prior studies examined only a small number of heterologous IDRs, limiting their analytical scope. In this study, we leveraged the evolutionary sequence divergence among yeast species and the genetic requirement of the Rim4 IDR in a nonessential process (sporulation) to investigate the sequences and properties that define IDR functions*.*

Although heterologous yeast prion IDRs failed to rescue Rim4 function, most *RIM4* orthologs encoding vastly diverged IDR sequences fully complemented. Our results indicate that, like many other IDRs, the functions mediated by the Rim4 C-terminal IDR require specific sequence features. Unlike most structured regions, however, a wide range of sequence space can support those functions. Cross-species complementation despite extensive sequence divergence may be an exception rather than the rule. An example is the Crz1 transcription factor, whose IDR drives pulsatile nuclear import under calcium stress—a behavior linked to fitness [79]. When Crz1 IDRs from other *Saccharomyces* clade members are swapped into *S. cerevisiae*, pulsatility is maintained, but IDRs from more distantly related fungi fail to do so, even though they support pulsatility in their native species [19]. Chimeric IDR experiments identified a motif that restores pulsatile dynamics in *S. cerevisiae* to an otherwise inactive variant.

We identified two notable exceptions to our earlier hypothesis that amyloid-like assembly is strictly required for Rim4 function. M→A substitutions in the *S. cerevisiae* IDR disrupted meiotic entry without disrupting assembly. Conversely, V→R substitutions in the *T. caseinolytica* IDR partially restored meiotic entry but did not restore assembly. We speculate that these mutations affect the meiotic activator function of Rim4, which could be independent of assembly. This would be distinct from the repressor function, which requires formation of amyloid-like assemblies to bind to its RNA targets [35]. Several molecular models could explain how a single IDR mediates both activator and repressor roles. One possibility is state-dependent co-factor switching: in its monomeric form, Rim4 might recruit a translation-activating partner, whereas assembly into higher-order oligomers could favor association with a repressive factor. This mechanism is reminiscent of *Drosophila* Orb2 (CPEB), which as a monomer binds a deadenylase to repress translation but, upon assembly, switches to recruit a poly(A) polymerase and thereby activate translation [39]. An alternative framework invokes binding site-dependent regulation. Our prior work showed that Rim4 binding to the 5′ UTR is both necessary and sufficient for repression, and relocating that site abolishes repression without preventing binding [35]. When Rim4 assembles at the 5′ UTR, it may sterically obscure the cap structure and block recruitment of the initiation complex, thereby enforcing repression. In contrast, binding in the 3′ UTR could boost translation via a biophysical “vectorial channeling” mechanism, as proposed by Gu and colleagues [80]: when assemblies form at the 3′ UTR, ribosomes cannot enter the assembly, restricting the post-termination small subunit’s diffusion along the transcript and channeling it back to the 5′ cap of the same mRNA, thereby enhancing ribosome recycling and boosting translation. In contrast, 5′ UTR binding does not promote channeling: after termination, subunits can freely diffuse away from the cap, lowering the chance of re-initiation on the same mRNA.

**Fig 7:**
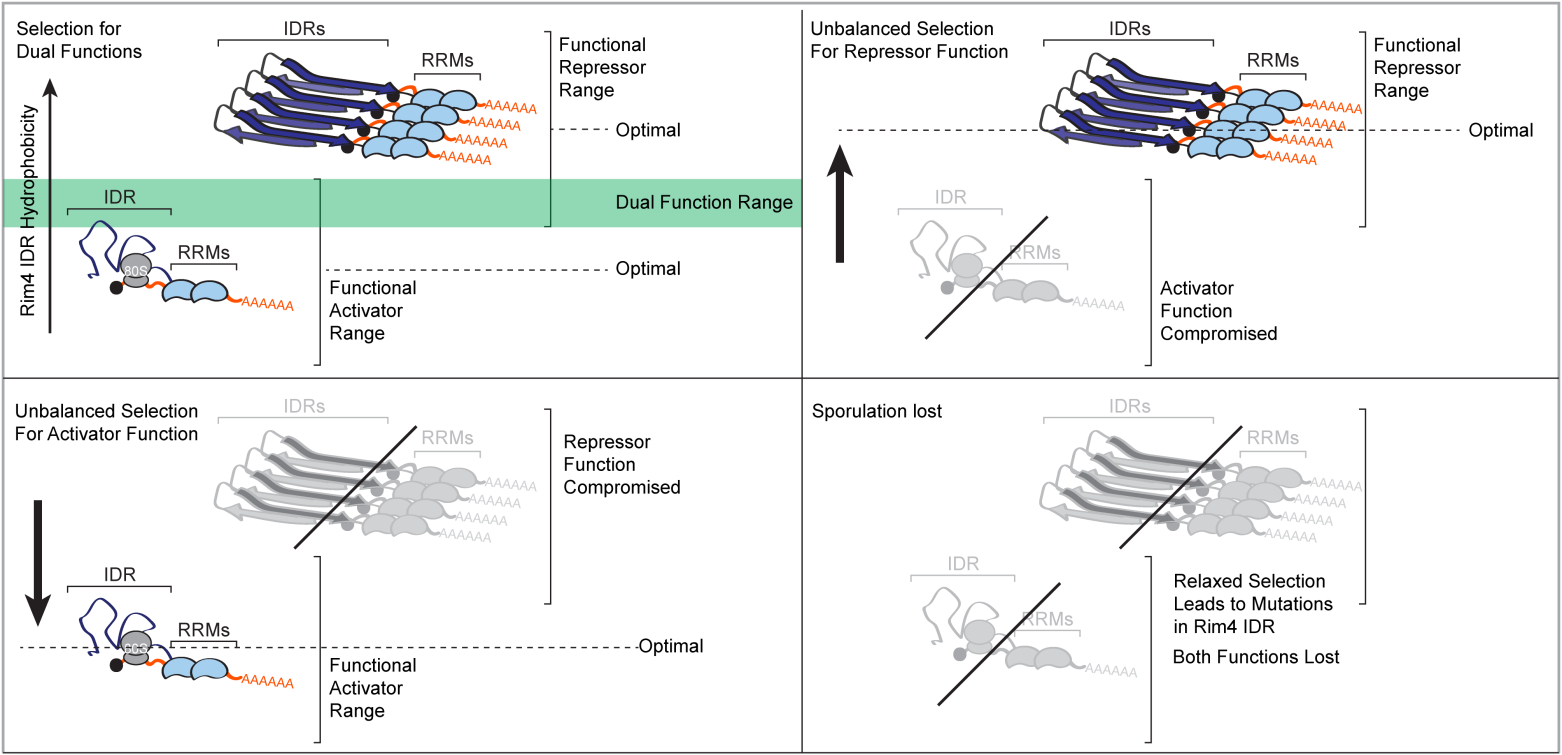
Evolutionary tuning of Rim4 intrinsically disordered region (IDR) hydrophobicity governs its dual functions. Schematic model showing how natural selection on the hydrophobicity of the Rim4 IDR could govern its ability to act as both a translational activator and repressor. Each function requires IDR hydrophobicity within a distinct, partially overlapping range. In *Saccharomyces cerevisiae* and fully complementing orthologs, hydrophobicity resides in the intersection of both optimal windows. Directional selection toward one function’s optimum—such as stronger repressor activity—shifts hydrophobicity out of the activator window and impairs that activity. Upon loss of sporulation, selective constraints on both functions are relaxed, allowing hydrophobicity (and consequently both activities) to drift and decay.

Our data are consistent with a model by which multiple nonoverlapping selective pressures influence sequence divergence of the *RIM4* IDR, and the early activator and later repressor functions of Rim4 could be under different evolutionary constraints (Fig 7). If the two IDR functions operate within even slightly different (overlapping) feature constraints, then increased selection for one function could compromise the other over evolutionary time. For example, during meiosis, an evolutionary arms race unfolds between LTR retrotransposons, which aim to proliferate, and the host defense function of Rim4, which suppresses their proliferation [32]. This conflict could drive selection to favor Rim4’s repressor function. If the repressor function operates optimally at a higher hydrophobicity than the activator function, this selective pressure may eventually compromise the activator function. Dual functions of IDRs are not unique to Rim4 [20,81]. For example, the IDR of the yeast transcription factor Msn2 supports both sequence binding specificity and transcriptional activation [20,82]. Notably, the transcriptional activator function of the Msn2 IDR appears to have diverged more rapidly (as early as 100 MYA) compared to the functions of the Rim4 IDR.

The assembly behavior of IDR-containing proteins is often influenced by temperature, particularly in sessile organisms such as plants and yeasts, which must rapidly react to their environment without the luxury of voluntary movement [16,83–86]. The ecological niche where a yeast exists could therefore influence *RIM4* IDR evolution, and Rim4 itself has been proposed to act as a thermosensor [87]. For example, *T. caseinolytica* is found in rotting cacti in the Sonoran Desert and thus may be adapted to higher temperatures. Given that the hydrophobic effect is stronger at higher temperatures and that decreased Rim4 hydrophobicity (paradoxically) leads to greater stability, the *T. caseinolytica* Rim4 IDR may utilize increased hydrophobicity to protect Rim4 function in the desert. In contrast, yeasts adapted to cold environments could exhibit lower Rim4 hydrophobicity to drive assembly and stability due to a weaker hydrophobic effect at lower temperatures.

Our findings support a model in which a defined hydrophobicity range is critical for Rim4 IDR function, reminiscent of how the yeast thermosensor Pab1 uses hydrophobicity to set its phase‐separation threshold: increasing hydrophobicity decreases the temperature required for demixing and vice versa [16]. A related principle governs heat‐induced condensate formation by the essential translation factor Ded1, whose reversible assemblies selectively repress translation of housekeeping mRNAs with long, structured 5′ UTRs while permitting the synthesis of stress‐response transcripts bearing shorter, unstructured leaders [88]. Ded1 orthologs from different yeast species display niche‐specific shifts in heat‐regulated assembly—changes traced to interplay between structured and unstructured regions—illustrating how ecological pressures tune IDR evolution [89]. In the filamentous fungus Ashbya gossypii, a similar mechanism operates: natural variation in the Whi3 IDR tunes the temperature at which Whi3 condensates form, thereby coordinating cell‐cycle entry and polarized growth with the organism’s preferred thermal range [21].

Unexpectedly, M→A substitutions in the Rim4 IDR led to the increased abundance of dysfunctional Rim4 assemblies. Hydrophobicity in the Rim4 IDR may alter solvent exposure and may reveal aggregation-prone hotspots—similar to how interactions in α-synuclein mask its central aggregation region [90]. In contrast, decreasing hydrophobicity in the *T. caseinolytica* Rim4 (by substituting V→R) IDR partially restored function. Although we did not observe formation of SDS-resistant assemblies in this mutant, arginine residues could mediate IDR interactions that drive assembly of nonSDS-resistant condensates [91]. The additional arginine may inhibit SDS-resistant assembly because their stacking generates electrostatic repulsion [92]. Overall, we propose that evolutionary conservation of Rim4 hydrophobicity maintains proper inter- and intra-molecular interactions while preventing dysfunctional aggregation.

Although traditional structural and sequence alignment-based approaches are often suboptimal for studying IDRs, innovative computational approaches have driven progress toward a unified understanding of their functions [23,24]. As research in the IDR field advances, researchers will increasingly rely on advanced computational strategies, including machine learning and artificial intelligence, to study these regions. Deep learning models, for instance, have been trained against continuous or distributed sequence features to accurately predict IDRs [93] and to functionally distinguish groups based on sequence features [27]. A comprehensive understanding of IDR sequence–function relationships will likely require integrating deep learning approaches with functional and quantitative genetic complementation data. However, machine learning techniques typically demand vast amounts of training data, and generating such complementation datasets remains a major challenge. One approach is to assay random CDS, such as those from a cDNA library, for IDR function using a quantitative functional readout. Using Rim4 as an example, a library of IDR chimeras could be generated and assessed for complementation using a heteroallele assay for meiotic recombination. Although these methods may seem low throughput by current standards, they will be crucial for achieving a systematic understanding of IDRs, which constitute 30%–50% of protein regions.

## Methods

### Yeast strain construction

All strains used (S2 Table) were derived from *S. cerevisiae* SK1 background using standard transformation methods, crosses, and dissections. All modifications were confirmed by PCR and sequencing and confirmed transformants were backcrossed with wild type before mating with strains containing desired constructs. All modifications of *RIM4* are markerless and generated by CRISPR/Cas9. As a starting point for all *RIM4* modification strains, we substituted the *RIM4* CDS with the **C. albicans* URA3* cassette. We then transformed this Ura+ strain with a repair template and a pCas plasmid containing the sequence 5′-ACCACCAACCAAGAGCCAAG-3′ to target the *URA3* CDS. Repair templates were some variation of the following: the *S. cerevisiae RIM4* promoter fused to an orthologous or mutant *RIM4*, fused to a V5 epitope tag. Orthologous *RIM4* sequences were amplified from the corresponding yeast strains which were acquired from the NRRL. Because *C. albicans* decodes CUG as serine rather than leucine, we changed every CUG codon to UCG so that, when expressed in *S. cerevisiae*, the protein sequence matches the native *C. albicans* sequence.

### Growth conditions

Cells were grown at 30°C in liquid culture with shaking. To induce synchronous meiosis, strains were inoculated in YEPD (1% yeast extract, 2% peptone, and 2% dextrose) at noon and grown for ~24 h. At noon of the following day, cells were diluted in BYTA (1% yeast extract, 2% tryptone, 1% potassium acetate, 50 mM potassium phthalate) to an OD_600_ of 0.3 and grown overnight. The following morning, cells were washed with water and resuspended in SPO medium (0.3% potassium acetate, pH 7.0, 0.02% raffinose) at OD_600_ of 1.8 and grown with shaking at 30°C. *pGAL-NDT80, GAL4.ER* strains were released from G2 arrest by the addition of 1 μM β-estradiol at 6 h.

To initiate protein turnover assays, cycloheximide was added to a final concentration of 200 μg/mL after 4 h of growth in SPO at 30°C, and 3.6 OD units of cells were collected every 30 min for 2.5 h thereafter. To assay meiotic recombination frequency, cells were grown in SPO medium for 24 h at 30°C, and 3.6 OD units of cells were incubated on plates lacking histidine for 48 h prior to colony counting.

### Meiotic progression analysis

0.81 OD units of cells were fixed in 3.7% formaldehyde overnight at 4°C. Cells were permeabilized with 1% Triton-X and mounted in 0.1 M KPO_4_ [pH 7.5], 1.2 M sorbitol with DAPI. Images were acquired using SoftWoRx (Cytiva) at 100× magnification (Olympus UPLXAPO, numerical aperture 1.45) using a DeltaVision microscope (GE Healthcare) equipped with an EDGE sCMOS 5.5 camera at room temperature (∼25°C) and analyzed using FIJI (ImageJ) software. Cells containing one distinct nucleus within the cell wall were classified as uninucleate. Dispersed spores were differentiated from uninucleate cells by checking if the nucleus occupied more than half of the area within the cell wall. Cells containing two distinct nuclei were classified as binucleate, and tetrads and cells containing more than two distinct nuclei were classified as multinucleate. Cells in anaphase I were scored as dinucleate. Images were acquired at 100× magnification with a DeltaVision microscope (GE Healthcare). A max intensity projection of 10 (0.2 μm) z-stacks was used. For live-cell epifluorescence imaging in meiosis, after 4 h of growth in SPO at 30°C, the cells were loaded onto a microfluidics chip (Cell Asic), stage positions were established, and cells were imaged every 5 min for 10 h [94]. Exposure conditions were as follows: FITC 100%T, 0.01 s; mCherry 10%T, 0.01 s; 3 z-stacks at 0.2 μm spacing. A reference image was also taken (POL 10%T, 0.01 s). A max intensity projection was used for analysis.

### Sodium dodecyl sulfate-polyacrylamide gel electrophoresis (SDS-PAGE) and immunoblots

7.2 OD units of cells were resuspended in 5% trichloroacetic acid (TCA, Fisher Chemical), washed with acetone, and air‐dried. SDS-PAGE lysis buffer (1× TE (10 mM Tris HCl [pH 8], 1 mM EDTA), 1× Halt Protease and Phosphatase Inhibitor Cocktail (Thermo Scientific), 2.75 mM DTT, 10 mM Tris HCl [pH 11]) and acid-washed glass beads (425–600 μm dia.) were added to the pellets. Pellets were processed in a FastPrep‐24 Homogenizer (MP Biomedical) machine for 45 s at 6.5 m/s. 3× SDS-PAGE loading buffer (187 mM Tris HCl [pH 6.8], 30% glycerol, 9% SDS, 6% β‐mercaptoethanol, 0.05% Bromophenol Blue) was added to pellets, and pH was adjusted with Tris HCl [pH 11]. Samples were boiled for 5 min and spun for 5 min at 16,000*g*.

SDS-PAGE was performed with BioRad materials in Criterion TGX Precast Gradient Gels 4%–15% with Precision Plus Protein Dual Color Standard as the ladder. Proteins were transferred to 0.45 μm nitrocellulose with a Trans‐Blot Turbo System using Trans‐Blot Turbo Transfer Stacks and 1× Trans‐Blot Turbo Transfer Buffer with 20% ethanol. Total protein was visualized using Ponceau S staining solution (Thermo Fisher) to confirm transfer. Membranes were blocked in 3% milk 1× TBS‐T (10 mM Trizma Base, 150 mM NaCl, 0.1% Tween 20, [pH 7.6]) for 1 h at room temperature.

Antibodies and dilutions used to blot constructs tagged with V5 and Pgk1 are listed. Membranes were visualized after an antibody incubation with the Amersham ECL Prime Western Blotting Detection Reagent (Cytiva) and an Amersham Imager 600 (GE Healthcare). α-Pgk1 (Thermo Fisher, mouse monoclonal, RRID:AB_2532235) was used at 1:20,000, and α-v5 (Thermo Fisher, mouse monoclonal, RRID:AB_2556564) was used at 1:2,000. An α-mouse HRP-conjugated secondary antibody (Cytiva) was used at 1:10,000. Signal was visualized using ECL prime chemiluminescence substrate (Cytiva), and acquisition of chemiluminescence images was conducted using an Amersham Imager 600 (GE Healthcare). At least three exposures were taken for each experiment to ensure that our signal did not saturate and that we were in the linear range of the instrument.

### Semi‐denaturing detergent agarose electrophoresis (SDD‐AGE)

9 OD units of cells were snap frozen and stored at −80°C. Protocol was performed at 4°C unless otherwise indicated. Cell pellets were resuspended in SDD‐AGE lysis buffer (100 mM Tris HCl pH 8.0, 20 mM NaCl, 2 mM MgCl_2_, 1% Triton‐X, 50 mM β‐mercaptoethanol, 2× Halt Protease and Phosphatase Inhibitor Cocktail (Thermo Scientific)) and zirconia beads (0.5 mm dia. Zirconia/Silica, BioSpec Products) at room temperature and processed in a FastPrep‐24 Homogenizer machine for 45 s at 6.5 m/s. Protein extracts were spun at 2,500*g* for 10 min. Supernatant was transferred to new tubes and spun at the same speed for 5 min. 4× SDD‐AGE loading buffer (0.5× TAE, 20% glycerol, 8% SDS, and 0.05% bromophenol blue, where 10× TAE is 0.4 M Trizma Base, 1.14% glacial acetic acid, 10 mM EDTA) was added to supernatant, and extracts were incubated for 10 min at room temperature. Extracts were loaded onto a 1.7% agarose gel in 0.5× TAES (0.5× TAE with 0.1% SDS) and were run at 25 volts overnight at 4°C with buffer recirculation. Protein was transferred to an Amersham Protran 0.45 μm nitrocellulose membrane (Cytiva) with Blotting Paper 703 (VWR) soaked in 1× TBS (10 mM Trizma Base, 150 mM NaCl) and Pro‐Series wipers via capillary action at room temperature.

### Synteny analysis, multiple sequence alignments, and phylogenetic analysis

*RIM4* ortholog candidates were generated from BLAST using *S. cerevisiae RIM4* amino acid sequence. Synteny was determined by analyzing the Yeast Gene Order Browser, the National Center for Biotechnology Information (NCBI), and through manual confirmation [95,96]. Orthologous *RIM4* sequences were aligned using COBALT [56]. Evolutionary distances between species were estimated using Shen and colleagues (2018) [55]. Phylogenetic trees were generated using PhyloT, visualized in an interactive tree of life (iTOL), and are based on NCBI taxonomy [58].

### IDR feature analysis

14-3-3 protein binding sites were predicted using 14-3-3-Pred [71]. SHD and SCD were calculated as shown in original publications (Eq. 14 of Sawle and Ghosh (2015) [59]; Eq. 4 of Zheng and colleagues (2020) [60]). Sequence disorder score was calculated using PLAAC [66], IUPred [67,68], and Metapredict [69]. Disorder probability of each amino acid residue was averaged in IUPred and Metapredict to acquire a final score. Sequence radius of gyration and end-to-end distance were predicted using ALBATROSS [75].

### Northern blots

3.6 OD units of cells were pelleted and snap frozen. Cell pellets were resuspended in TES buffer (10 mM Tris HCl pH 7.5, 10 mM EDTA, 0.5% SDS), zirconia beads, and acid‐Phenol:Chloroform [pH 4.5] (with IAA 125:24:1, Ambion), and incubated for 30 min at 65°C with 1,000 rpm shaking. 1 ml 100% ethanol and 40 μl 3 M NaAc [pH 5.5] were added to the supernatant, and supernatants were precipitated overnight at −20°C. RNA was then pelleted, washed with 80% ethanol, and air-dried overnight.

RNA was run in denaturing 1.3% agarose gels containing 1× MOPS (where 10× MOPS is 0.2 M MOPS, 0.05 M NaAc, 0.01 M EDTA, [pH 7]) and 16.6% formaldehyde. RNA was transferred to an Amersham Hybond‐N+ membrane (GE Healthcare), soaked in 10× SSC (1.5 M NaCl, 0.15 M Trisodium citrate dihydrate) through capillary transfer for >48 h at room temperature. Membranes were crosslinked with 120,000 μJ/cm^2^ in a Spectrolinker XL‐1000 UV crosslinker (Spectronics corporation) and stained with a Methylene Blue solution (0.03% methylene blue, 0.4 M NaAc, pH 5.5) to visualize ribosomal RNA. Membranes were then incubated in 18 ml ULTRAhyb-Oligo buffer (Invitrogen) at 42°C rotating for 1 h. We prepared DNA oligonucleotide probes designed to recognize *CLB3*:

5′ biotin-CGCTTTCTCCGTCGTCTTCAACGGGTTCCTCTTGTTCCCTGTCCG

Membranes were incubated with 18 ml ULTRAhyb-Oligo buffer and 5 nM DNA oligonucleotide probe at 42°C, rotating overnight, washed twice with low stringency wash (2× SSC, 1% SDS) and once with high stringency wash (0.1× SSC, 1% SDS) at 42°C, incubated in blocking buffer (Thermo Scientific) for 15 min at room temperature. Membranes were visualized after a Stabilized Streptavidin-Horseradish Peroxidase Conjugate incubation with the Amersham ECL Prime Western Blotting Detection Reagent (Cytiva) and an Amersham Imager 600 (GE Healthcare).

### Statistics and reproducibility

All statistical analyses were conducted using Prism (V10, GraphPad). Unless indicated otherwise, statistical significance was determined by Student *t* test, multiple *t* tests with correction for multiple comparisons, one-way ANOVA with correction for multiple comparisons, or Mann–Whitney test, as indicated in figure legends. The data met the assumptions of the statistical tests used. No statistical method was used to predetermine sample size, but our sample sizes are similar to those reported in previous publications. No data were excluded from the analyses.

## Supporting information

S1 Fig Heterologous yeast PrLD-containing IDRs fail to complement Rim4’s IDR. **(A)** Northern blot analysis of *IME2* mRNA during early meiosis. Diploid strains expressing wild type *RIM4*, the RRM-inactive *rim4-rrm* mutant (F139→L), *rim4Δ*, or the IDR truncation *rim4Δ204* (lacking the C-terminal 204 residues) were induced to sporulate at 30°C. Samples were collected at the indicated time points, and *rRNA* served as a loading control. **(B)** Diploids homozygous for *RNQ1-3V5* were either grown to log phase in rich medium (YPD) or induced to sporulate at 30°C (SPO) and grown for 3 h. Presence of Rnq1 SDS-resistant assemblies was tested using SDD-AGE. **(C)** Extended meiotic progression data for Fig 1B. Diploid strains were induced to sporulate at 30°C. Progression through meiotic divisions was by DAPI staining (*n* = 3 biological replicates). The data underlying this figure can be found in S1 Data.
(TIF)

S2 Fig Verification of *RIM4* ortholog candidates by syntenic analysis. Syntenic relationships surrounding candidate *RIM4* loci are presented alongside a phylogenetic tree of the analyzed yeast species. For each candidate locus, up to six overlapping syntenic genes are displayed. Parallel slashes indicate discontinuities where one or two genes have been omitted. Gene orientation is marked with arrows, and orthologous genes are shaded in the same colors.
(TIF)

S3 Fig The *RIM4* IDR is poorly conserved compared to its structured regions. **(A)** A multiple sequence alignment of *RIM4* orthologs is shown alongside a phylogenetic tree of the analyzed yeast species. Conserved residues are shaded based on their level of conservation, while nonconserved regions are highlighted in peach. **(B)** The distribution of aromatic residues and RRM domains is displayed next to a phylogenetic tree of the analyzed yeast species. RRM domains are shown in light blue, IDRs in gray, and aromatic residues (F, Y) are highlighted in green.
(TIF)

S4 Fig Meiotic progression of orthologous *RIM4* substitutions. **(A)** Extended meiotic progression data for Fig 3B. Diploid strains were induced to sporulate at 30°C. Progression through the meiotic divisions was determined by DAPI staining (*n* = 3 biological replicates). **(B)** Statistical analysis of meiotic complementation by orthologous *RIM4* IDRs. Left: Mann–Whitney U test comparing sporulation efficiency of complementing versus non‐complementing IDRs. Right: receiver‐operating characteristic (ROC) curve assessing how well IDR identity discriminates between the two groups. For both tests, the **Komagataella* phaffii* IDR was included in the complementing group based on its IDR substitution assay performance shown in Fig 4B. The data underlying this figure can be found in S1 Data.
(TIF)

S5 Fig Meiotic analysis of IDR chimeras. **(A)** Extended meiotic progression data for Fig 4B. Diploid strains were induced to sporulate at 30°C. Progression through the meiotic divisions was determined by DAPI staining (*n* = 3 biological replicates). **(B)** Live‐cell epifluorescence time‐lapse of diploid strains homozygous for *HTB1-mCherry* (nuclear marker, red) and C-terminal EGFP fusions to wild type Rim4, Rim4ΔIDR, or an IDR chimera (green). After 4 h of sporulation at 30°C, cells were loaded into a CellASIC microfluidics chamber and imaged every 5 min. The frames show the merged fluorescence channels overlaid on DIC. Scale bar, 5 μm. **(C)** Cycloheximide chase analysis of Rim4 stability during meiosis. At 4 h after transfer to sporulation medium, cycloheximide was added, and samples were collected every 30 min for 2.5 h. Homozygous IDR substitution strains were lysed, and Rim4 protein levels were analyzed by SDS-PAGE/ immunoblot with Pgk1 as a loading control. Data from 3 biological replicates are normalized to the abundance at the time of cycloheximide addition (*t* = 0) and fitted with a one-phase exponential decay curve. A Mann–Whitney test U was used to compare half-life of complementing versus noncomplementing IDRs. The data underlying this figure can be found in S1 Data.
(TIF)

S6 Fig Complementing Rim4 IDRs support meiotic progression, degradation kinetics, and translational repression. **(A–C)** Extended data for Fig 5. Strains harboring *NDT80-IN*, *GAL4.ER*, and *CLB3-3HA*, and were induced to sporulate at 30°C. At 6 h, cells were released from the G2 arrest. (A) The percentage (*n* = 100 cells for each time point) of metaphase I, anaphase I, metaphase II, and anaphase II cells was determined by tubulin IF and DAPI staining. (B) Single-cell Rim4 levels in cells in metaphase I, anaphase I, metaphase II, and anaphase II were determined by V5 IF (*n* = 50 cells per meiotic stage). (C) Immunoblot (Rim4, Clb3, and Pgk1) and northern blot (*CLB3* and *rRNA*) source data used for quantifications shown in Fig 5C. The data underlying this figure can be found in S1 Data.
(TIF)

S7 Fig Rim4 IDR chimeras form SDS-resistant assemblies that do not display heterotypic interference. **(A, B)** Homozygous diploids expressing Rim4 IDR chimeras were induced to sporulate at 30°C. (A) Six hours post-induction, cell lysates were analyzed by SDD-AGE to detect SDS-resistant Rim4 assemblies. (B) Parallel samples were boiled before SDD-AGE to test for Rim4 assemblies resistant to both heat and SDS. **(C)** Diagram of heterozygous *RIM4* strains containing one copy of *Saccharomyces cerevisiae RIM4* and one copy of an orthologous *RIM4* CDS. **(D, E)** Heterozygous strains were induced to sporulate at 30°C. (D) Meiotic progression was monitored by DAPI staining. (E) Presence of Rim4 SDS-resistant assemblies were determined by SDD-AGE, and total Rim4 levels were determined by SDS-PAGE/immunoblot. The data underlying this figure can be found in S1 Data.
(TIF)

S8 Fig Meiotic progression of IDR mutants. Detailed meiotic progression data for Fig 6B (A) and 6E (B). Diploid strains were induced to sporulate at 30°C. Progression through meiotic divisions was determined by DAPI staining of nuclei (*n* = 3 biological replicates). The data underlying this figure can be found in S1 Data.
(TIF)

S9 Fig Meiotic progression of regional V→R *T.* *caseinolytica* IDR mutants. **(A, B)** Extended data for Fig 6G. Diploid strains harboring regional V→R mutations (sequential thirds) were induced to sporulate at 30°C. (A) Progression through meiotic divisions was determined by DAPI staining of nuclei (*n* = 3 biological replicates). (B) Cells were lysed, and Rim4 protein levels were analyzed by SDS-PAGE/immunoblot with Pgk1 as a loading control. The data underlying this figure can be found in S1 Data.
(TIF)

S1 Table Biophysical and biochemical characterization of yeast Rim4 IDR orthologs. (XLSX)

S2 Table Strains used in this study. (XLSX)

S1 Data Data underlying all figures. (XLSX)

S2 Data Raw images. (ZIP)

## Abbreviations

CDS: coding sequence
IDR: intrinsically disordered region
MYA: million years ago
NCBI: National Center for Biotechnology Information
polyN: polyasparagine
PrLD: prion-like domain
RBP: RNA-binding protein
RRM: RNA recognition motif
SCD: sequence charge decoration
SDD-AGE: semi-denaturing detergent agarose gel electrophoresis
SDS-PAGE: sodium dodecyl sulfate-polyacrylamide gel electrophoresis
SHD: sequence hydropathy decoration
