# Supplementary figures and images for: Cross‐species rescue reveals sequence requirements for a rapidly evolving intrinsically disordered region
Source: PLoS Biol. 2025 Sep 25;23(9):e3003396. doi: 10.1371/journal.pbio.3003396 (PMC12483275; doi:10.1371/journal.pbio.3003396)

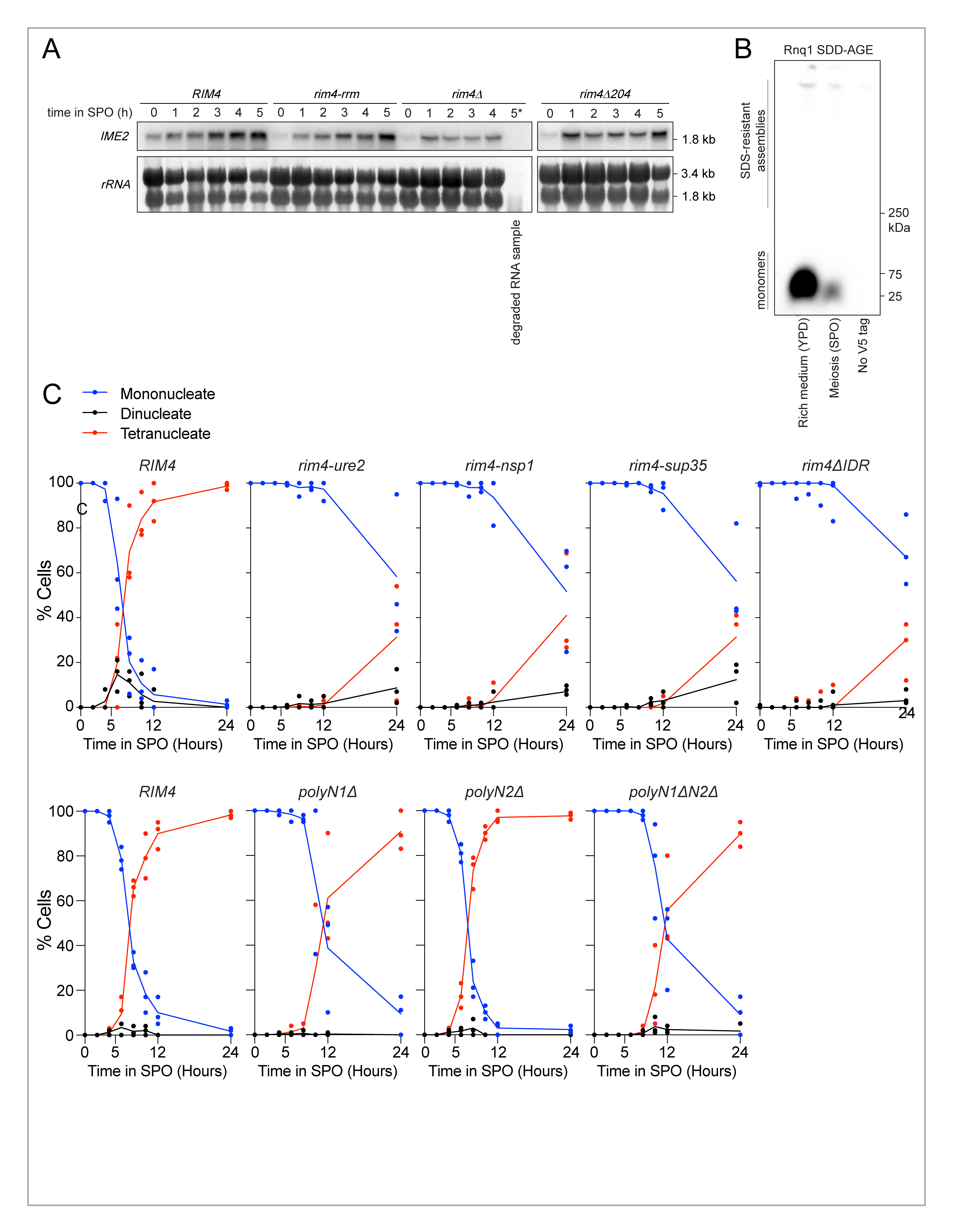

Supplement: S1 Fig — (A) Northern blot analysis of IME2 mRNA during early meiosis. Diploid strains expressing wild type RIM4, the RRM-inactive rim4-rrm mutant (F139→L), rim4Δ, or the IDR truncation rim4Δ204 (lacking the C-terminal 204 residues) were induced to sporulate at 30°C. Samples were collected at the indicated time points, and rRNA served as a loading control. (B) Diploids homozygous for RNQ1-3V5 were either grown to log phase in rich medium (YPD) or induced to sporulate at 30°C (SPO) and grown for 3 h. Presence of Rnq1 SDS-resistant assemblies was tested using SDD-AGE. (C) Extended meiotic progression data for Fig 1B. Diploid strains were induced to sporulate at 30°C. Progression through meiotic divisions was by DAPI staining (n = 3 biological replicates). The data underlying this figure can be found in S1 Data. (TIF) [file pbio.3003396.s001.tif]

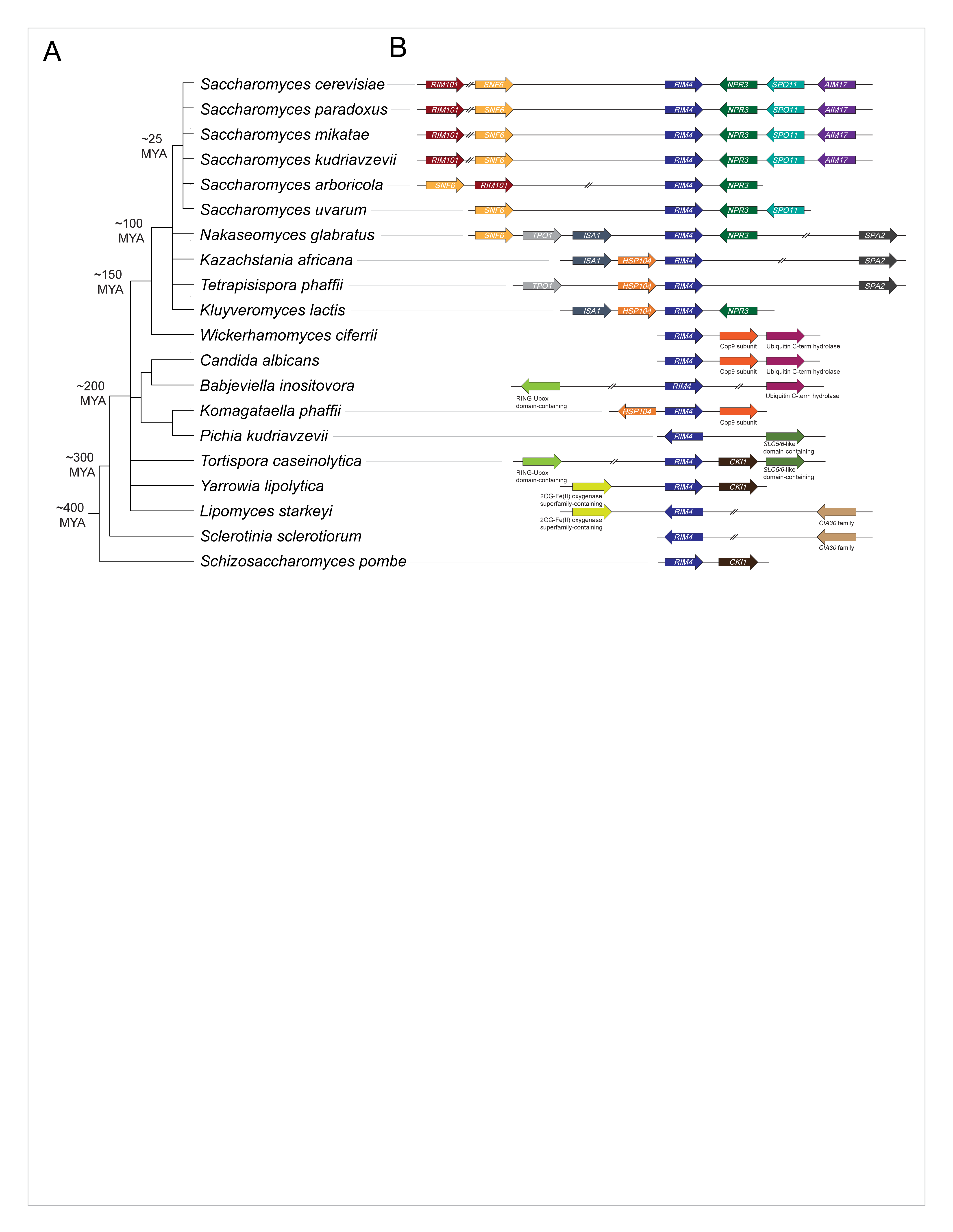

Supplement: S2 Fig — Syntenic relationships surrounding candidate RIM4 loci are presented alongside a phylogenetic tree of the analyzed yeast species. For each candidate locus, up to six overlapping syntenic genes are displayed. Parallel slashes indicate discontinuities where one or two genes have been omitted. Gene orientation is marked with arrows, and orthologous genes are shaded in the same colors. (TIF) [file pbio.3003396.s002.tif]

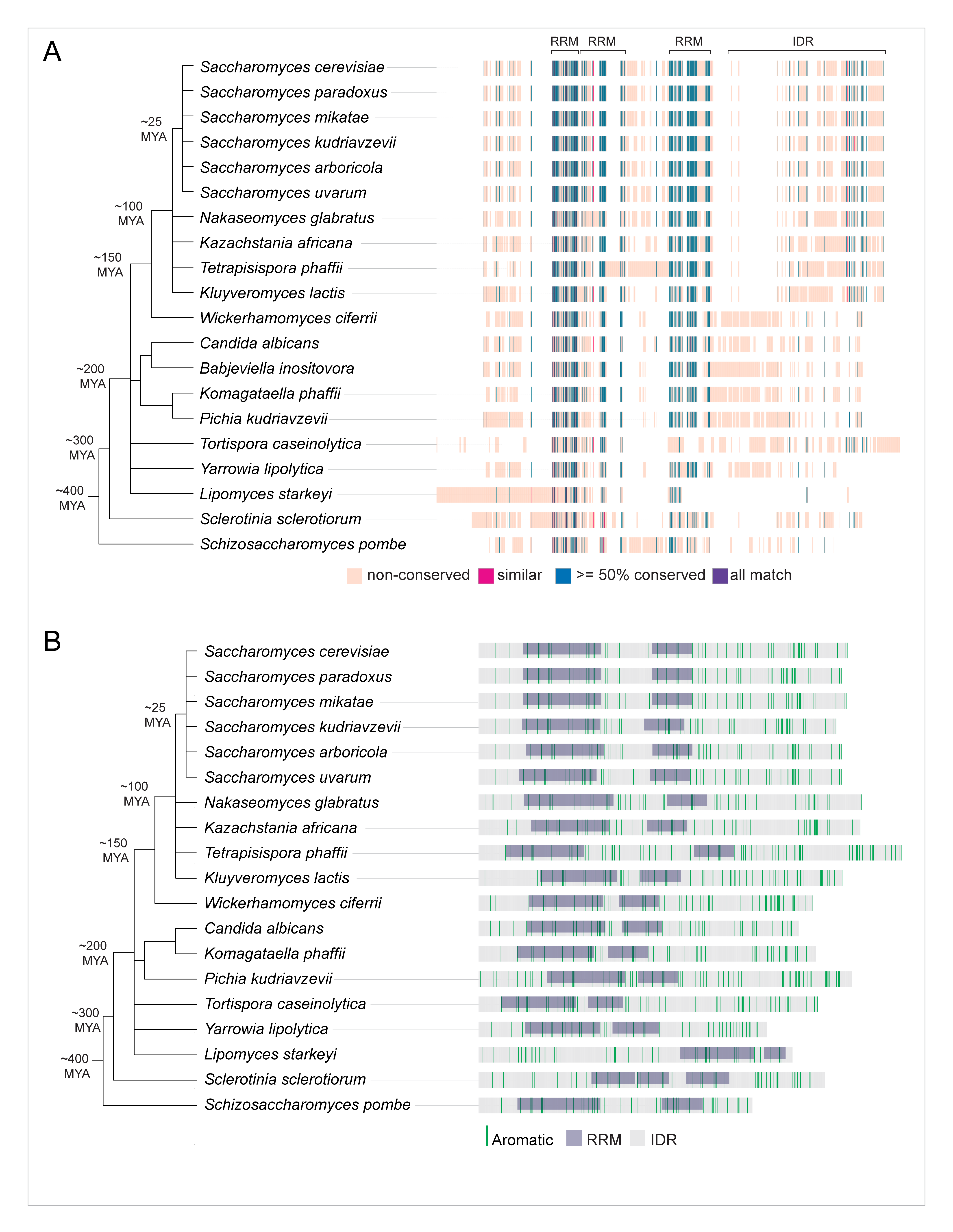

Supplement: S3 Fig — (A) A multiple sequence alignment of RIM4 orthologs is shown alongside a phylogenetic tree of the analyzed yeast species. Conserved residues are shaded based on their level of conservation, while nonconserved regions are highlighted in peach. (B) The distribution of aromatic residues and RRM domains is displayed next to a phylogenetic tree of the analyzed yeast species. RRM domains are shown in light blue, IDRs in gray, and aromatic residues (F, Y) are highlighted in green. (TIF) [file pbio.3003396.s003.tif]

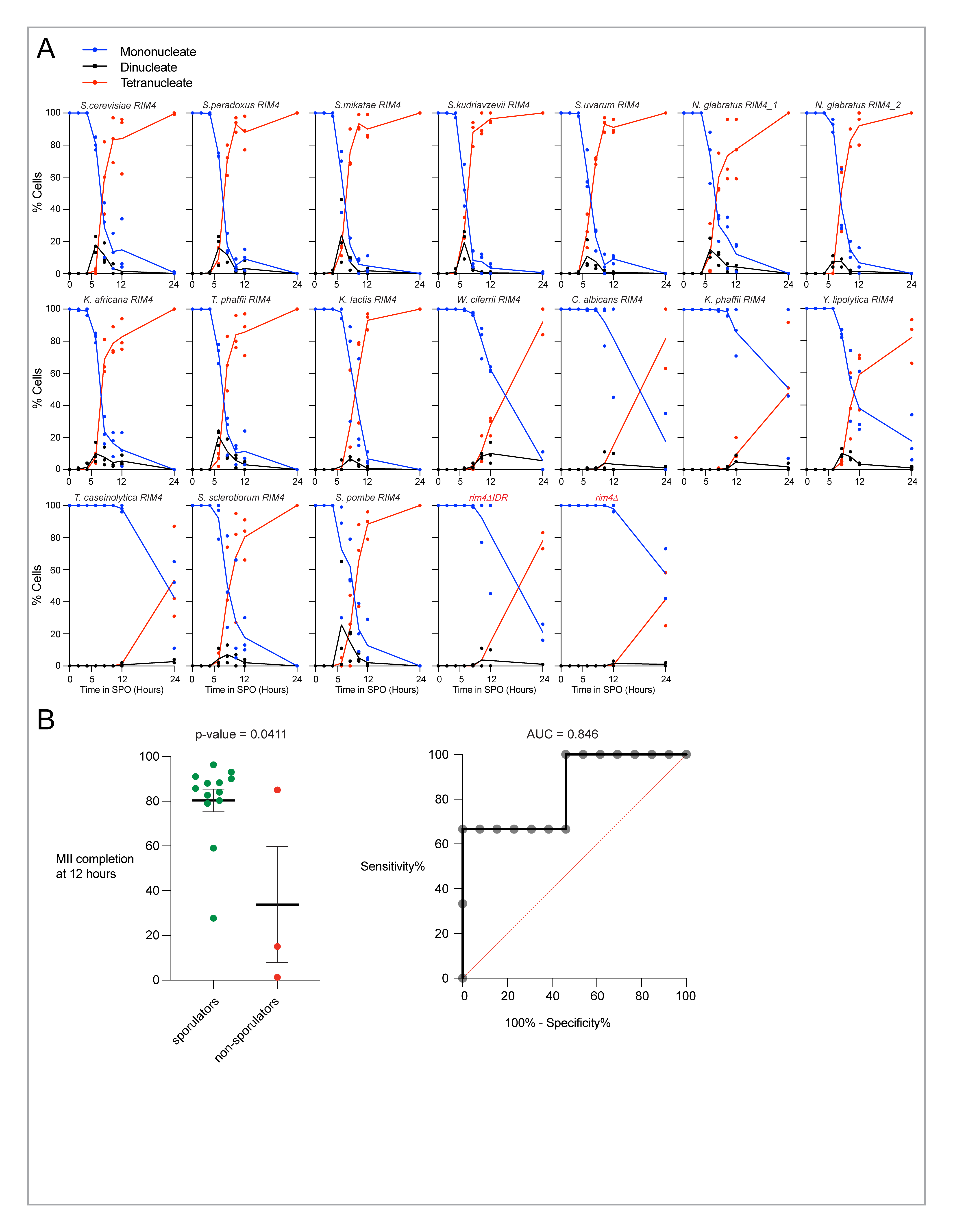

Supplement: S4 Fig — (A) Extended meiotic progression data for Fig 3B. Diploid strains were induced to sporulate at 30°C. Progression through the meiotic divisions was determined by DAPI staining (n = 3 biological replicates). (B) Statistical analysis of meiotic complementation by orthologous RIM4 IDRs. Left: Mann–Whitney U test comparing sporulation efficiency of complementing versus non‐complementing IDRs. Right: receiver‐operating characteristic (ROC) curve assessing how well IDR identity discriminates between the two groups. For both tests, the Komagataella phaffii IDR was included in the complementing group based on its IDR substitution assay performance shown in Fig 4B. The data underlying this figure can be found in S1 Data. (TIF) [file pbio.3003396.s004.tif]

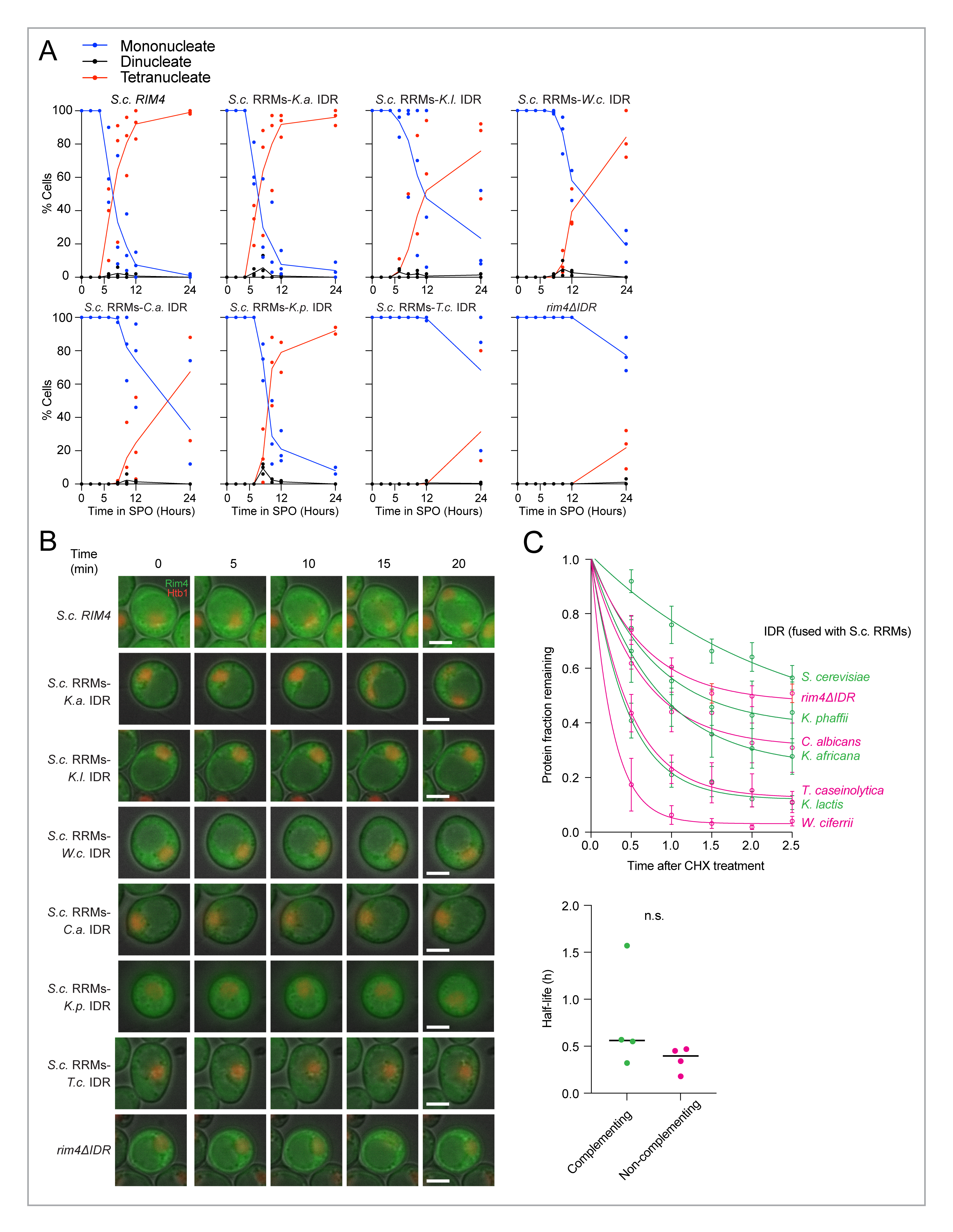

Supplement: S5 Fig — (A) Extended meiotic progression data for Fig 4B. Diploid strains were induced to sporulate at 30°C. Progression through the meiotic divisions was determined by DAPI staining (n = 3 biological replicates). (B) Live‐cell epifluorescence time‐lapse of diploid strains homozygous for HTB1-mCherry (nuclear marker, red) and C-terminal EGFP fusions to wild type Rim4, Rim4ΔIDR, or an IDR chimera (green). After 4 h of sporulation at 30°C, cells were loaded into a CellASIC microfluidics chamber and imaged every 5 min. The frames show the merged fluorescence channels overlaid on DIC. Scale bar, 5 μm. (C) Cycloheximide chase analysis of Rim4 stability during meiosis. At 4 h after transfer to sporulation medium, cycloheximide was added, and samples were collected every 30 min for 2.5 h. Homozygous IDR substitution strains were lysed, and Rim4 protein levels were analyzed by SDS-PAGE/ immunoblot with Pgk1 as a loading control. Data from 3 biological replicates are normalized to the abundance at the time of cycloheximide addition (t = 0) and fitted with a one-phase exponential decay curve. A Mann–Whitney test U was used to compare half-life of complementing versus noncomplementing IDRs. The data underlying this figure can be found in S1 Data. (TIF) [file pbio.3003396.s005.tif]

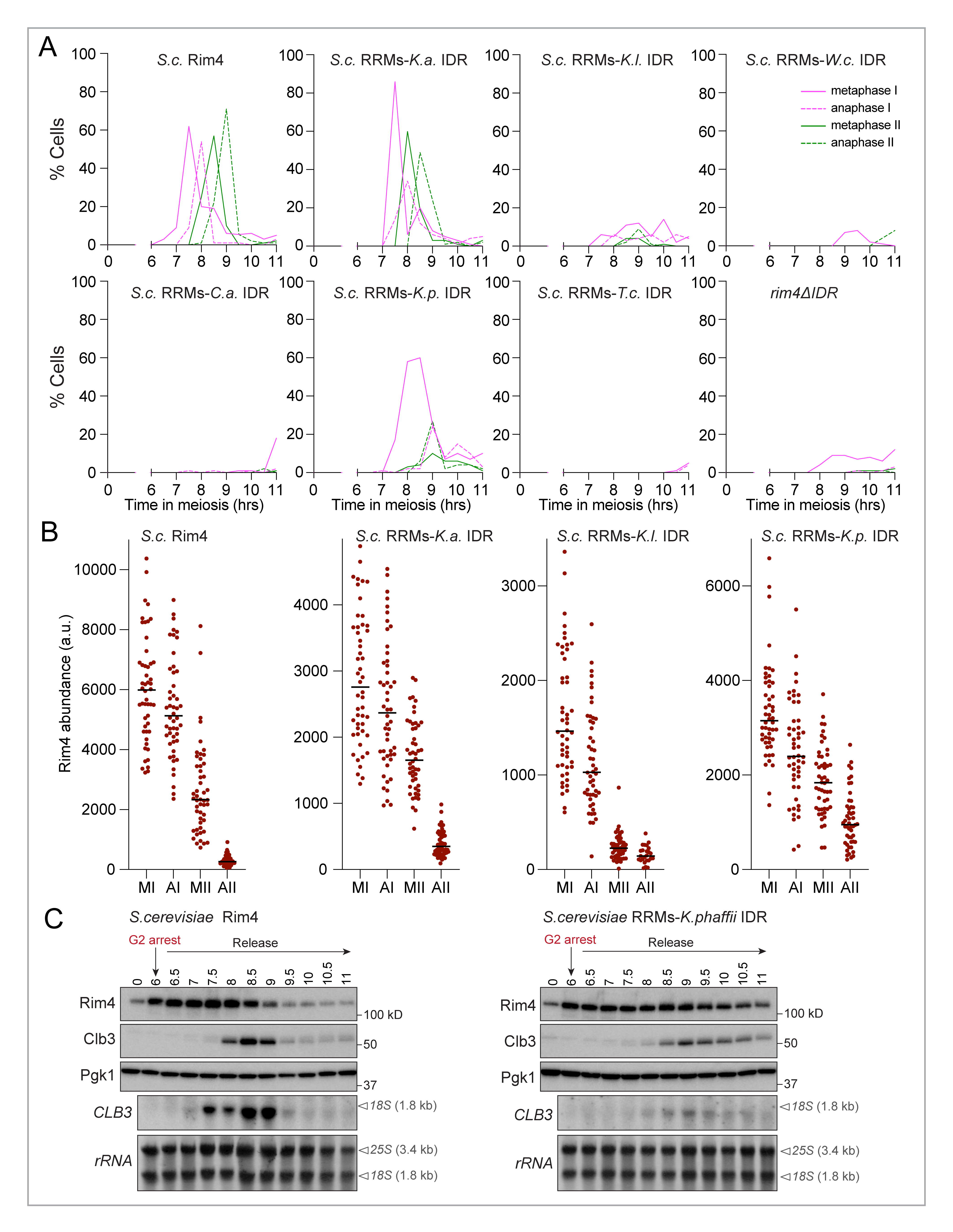

Supplement: S6 Fig — (A–C) Extended data for Fig 5. Strains harboring NDT80-IN, GAL4.ER, and CLB3-3HA, and were induced to sporulate at 30°C. At 6 h, cells were released from the G2 arrest. (A) The percentage (n = 100 cells for each time point) of metaphase I, anaphase I, metaphase II, and anaphase II cells was determined by tubulin IF and DAPI staining. (B) Single-cell Rim4 levels in cells in metaphase I, anaphase I, metaphase II, and anaphase II were determined by V5 IF (n = 50 cells per meiotic stage). (C) Immunoblot (Rim4, Clb3, and Pgk1) and northern blot (CLB3 and rRNA) source data used for quantifications shown in Fig 5C. The data underlying this figure can be found in S1 Data. (TIF) [file pbio.3003396.s006.tif]

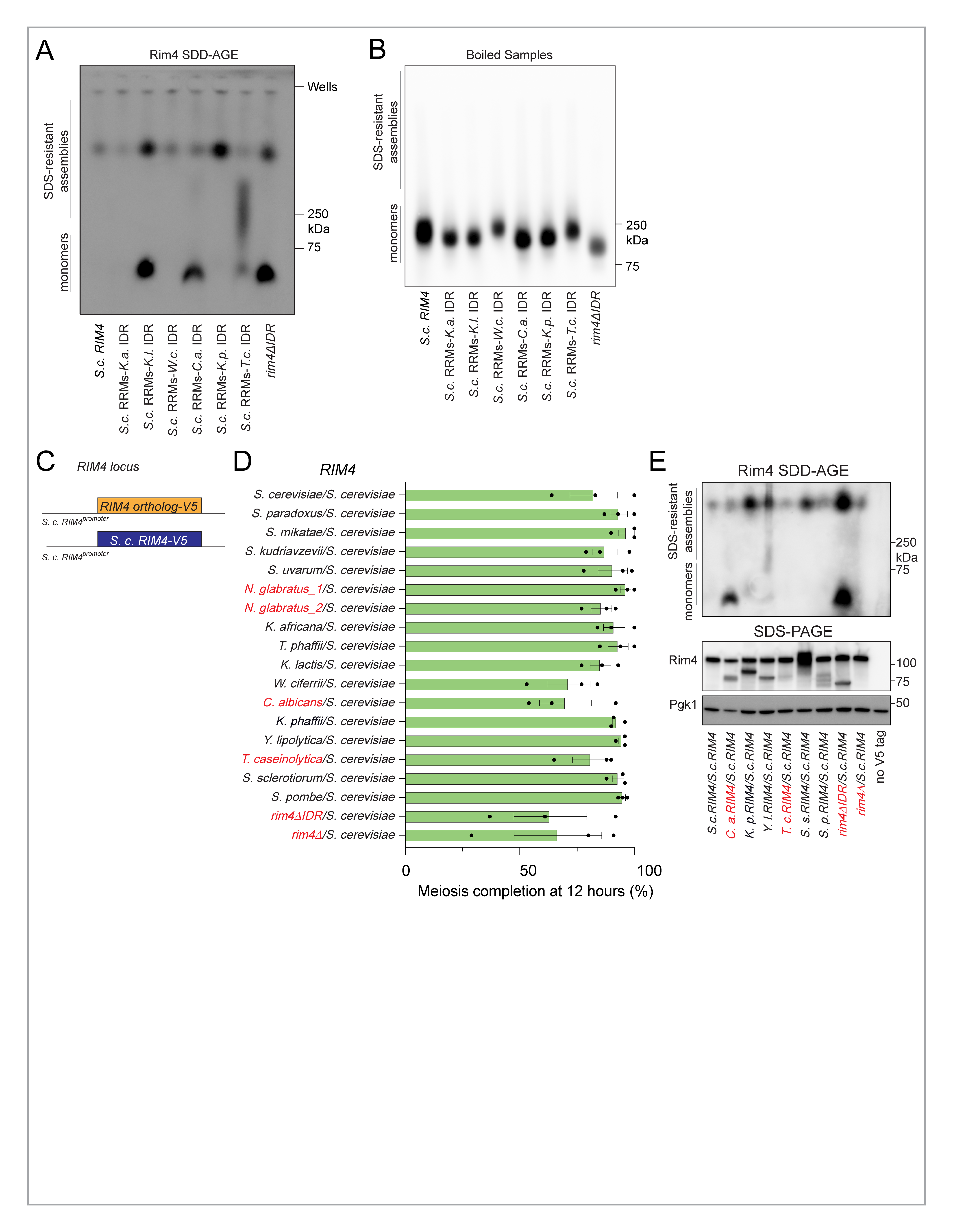

Supplement: S7 Fig — (A, B) Homozygous diploids expressing Rim4 IDR chimeras were induced to sporulate at 30°C. (A) Six hours post-induction, cell lysates were analyzed by SDD-AGE to detect SDS-resistant Rim4 assemblies. (B) Parallel samples were boiled before SDD-AGE to test for Rim4 assemblies resistant to both heat and SDS. (C) Diagram of heterozygous RIM4 strains containing one copy of Saccharomyces cerevisiae RIM4 and one copy of an orthologous RIM4 CDS. (D, E) Heterozygous strains were induced to sporulate at 30°C. (D) Meiotic progression was monitored by DAPI staining. (E) Presence of Rim4 SDS-resistant assemblies were determined by SDD-AGE, and total Rim4 levels were determined by SDS-PAGE/immunoblot. The data underlying this figure can be found in S1 Data. (TIF) [file pbio.3003396.s007.tif]

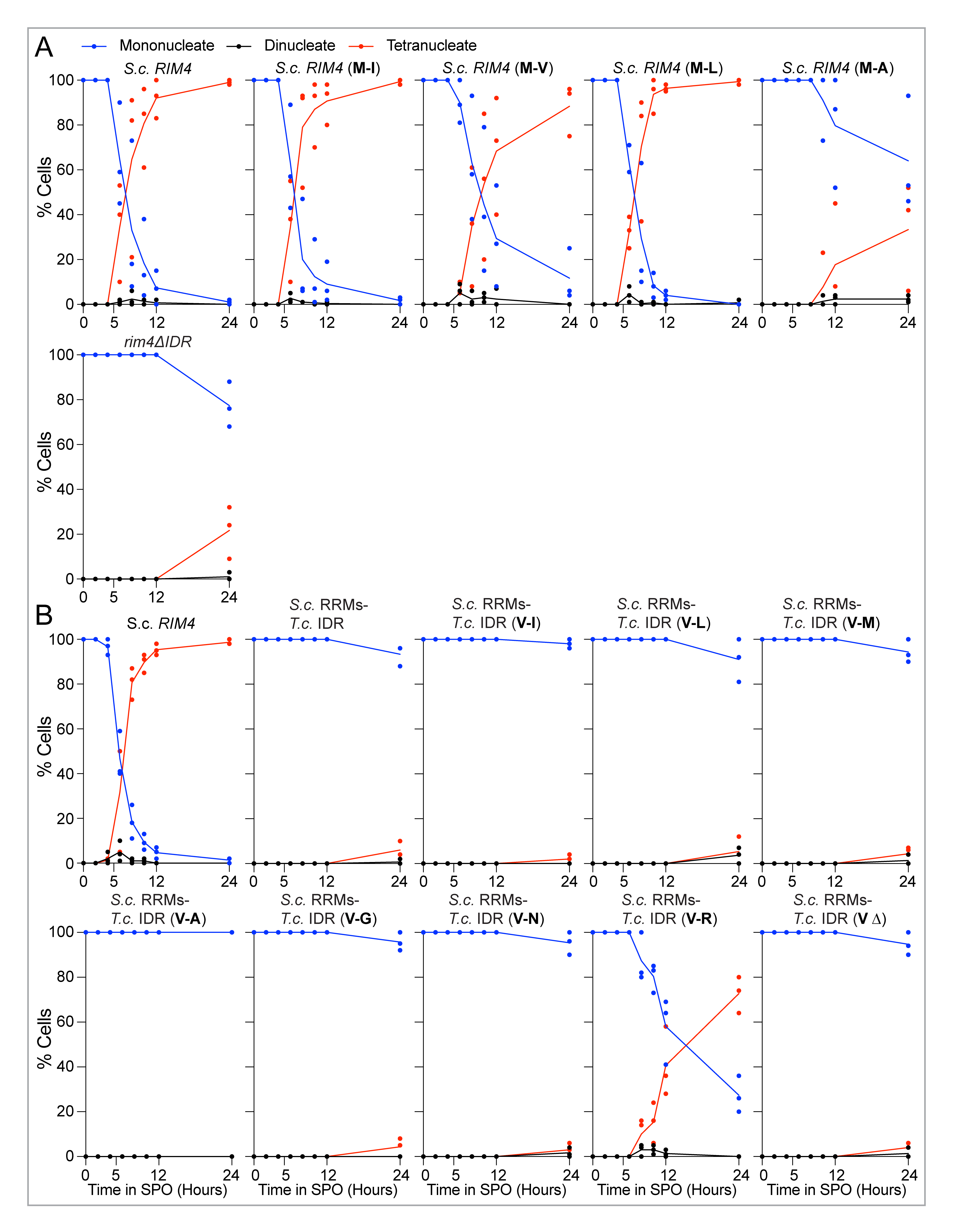

Supplement: S8 Fig — Detailed meiotic progression data for Fig 6B (A) and 6E (B). Diploid strains were induced to sporulate at 30°C. Progression through meiotic divisions was determined by DAPI staining of nuclei (n = 3 biological replicates). The data underlying this figure can be found in S1 Data. (TIF) [file pbio.3003396.s008.tif]

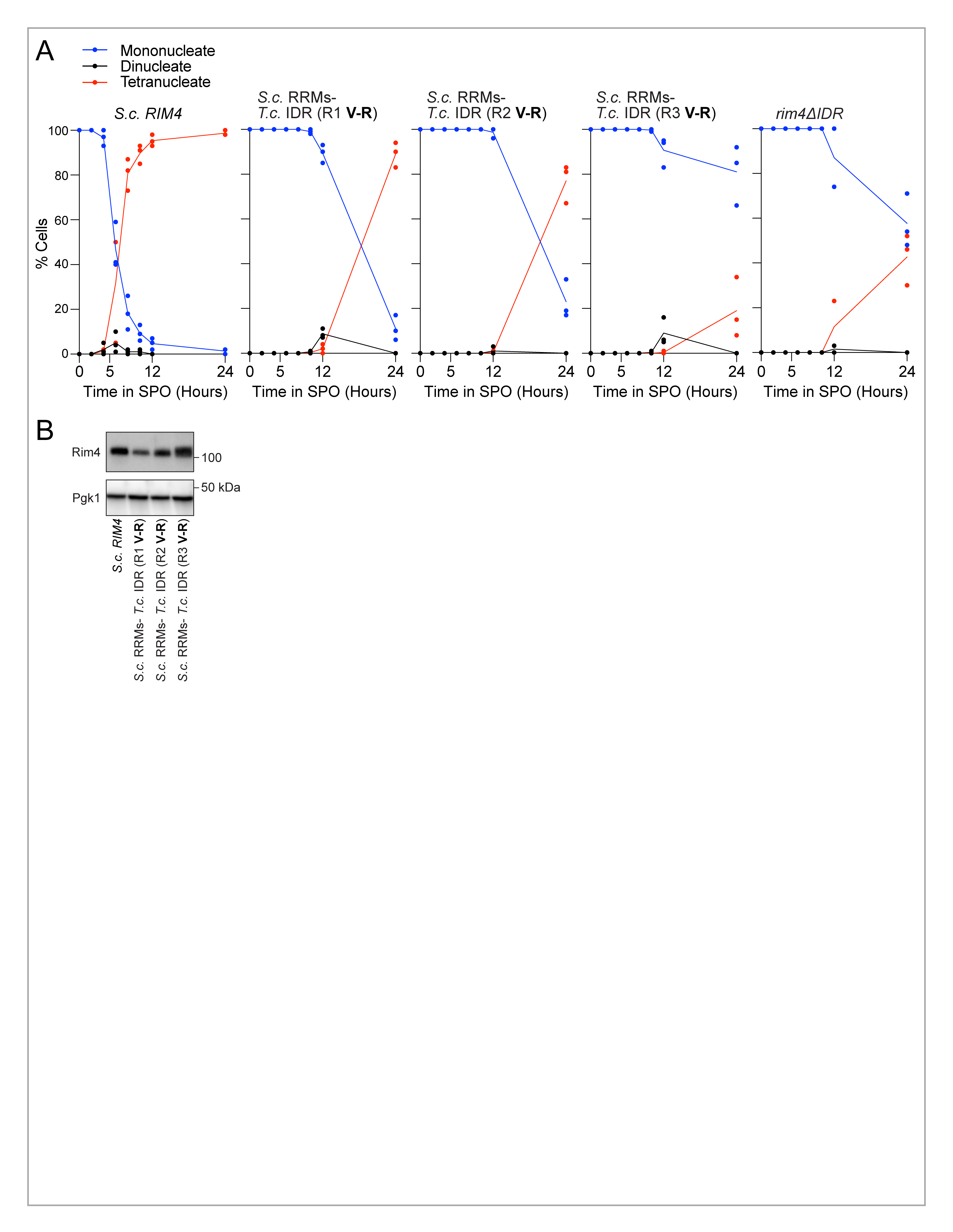

Supplement: S9 Fig — (A, B) Extended data for Fig 6G. Diploid strains harboring regional V→R mutations (sequential thirds) were induced to sporulate at 30°C. (A) Progression through meiotic divisions was determined by DAPI staining of nuclei (n = 3 biological replicates). (B) Cells were lysed, and Rim4 protein levels were analyzed by SDS-PAGE/immunoblot with Pgk1 as a loading control. The data underlying this figure can be found in S1 Data. (TIF) [file pbio.3003396.s009.tif]

1C

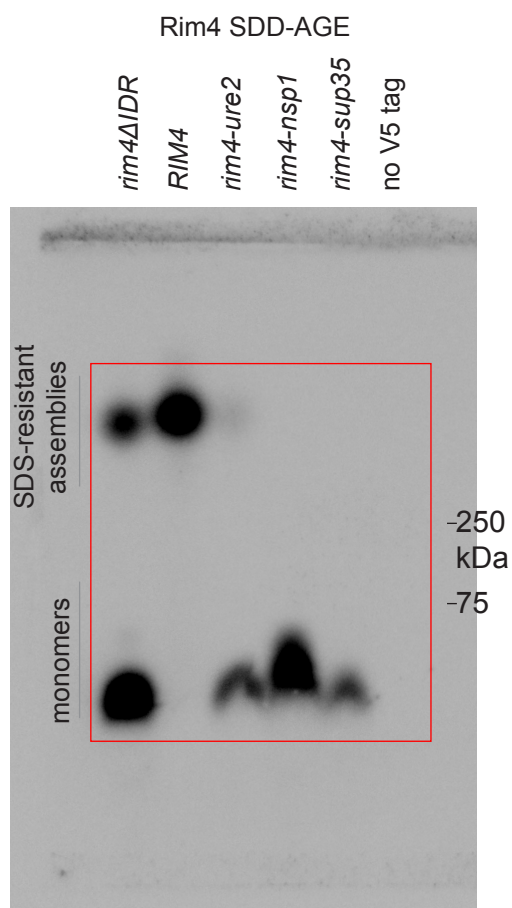

Rim4 SDS-PAGE

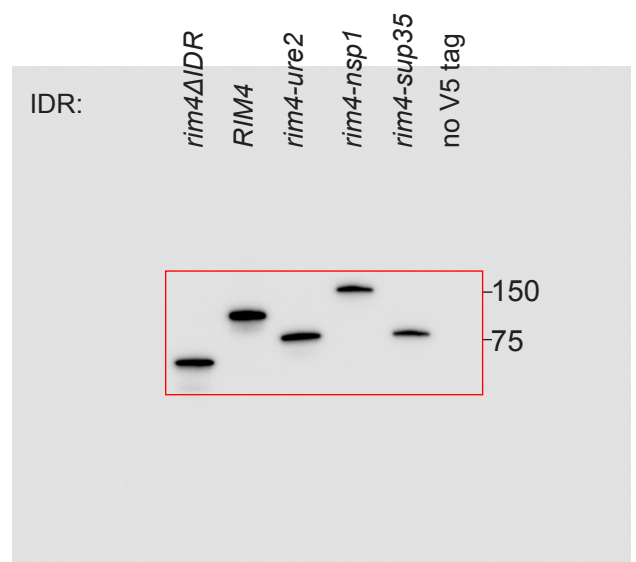

1F

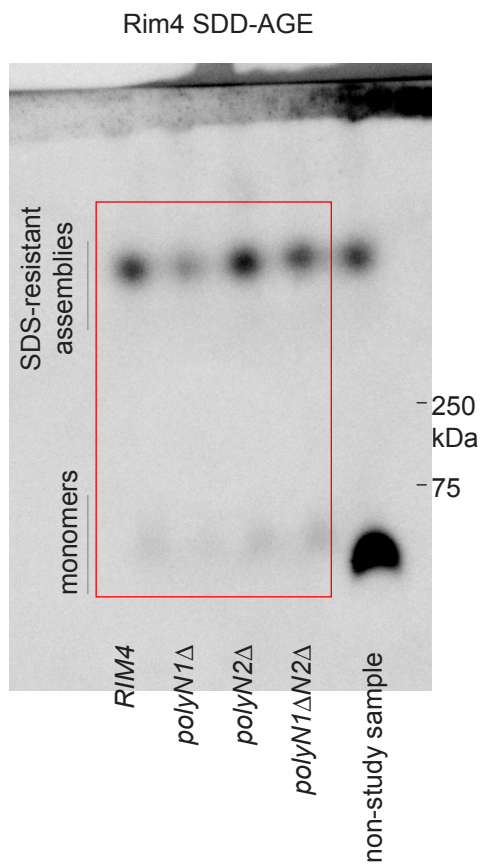

Rim4 SDS-PAGE

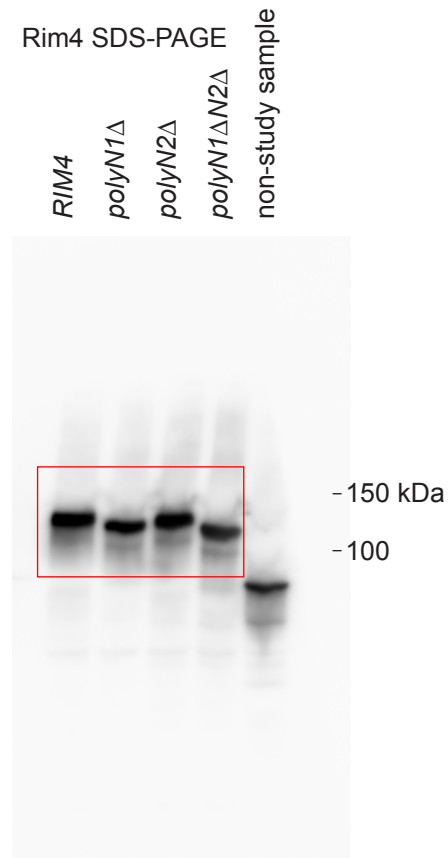

Pgl1 SDS-PAGE

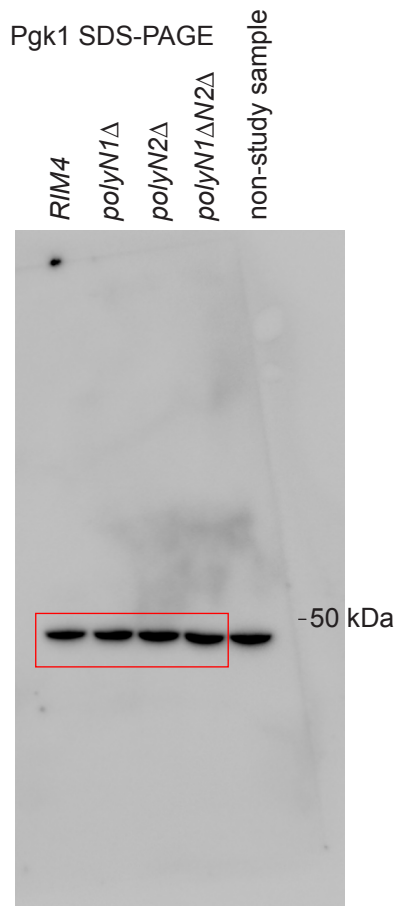

Supplement: S2 Data — (ZIP) [file pbio.3003396.s013.zip › S1_Raw_Images/Raw_Images_Fig1.pdf]

Rim4 SDS-PAGE

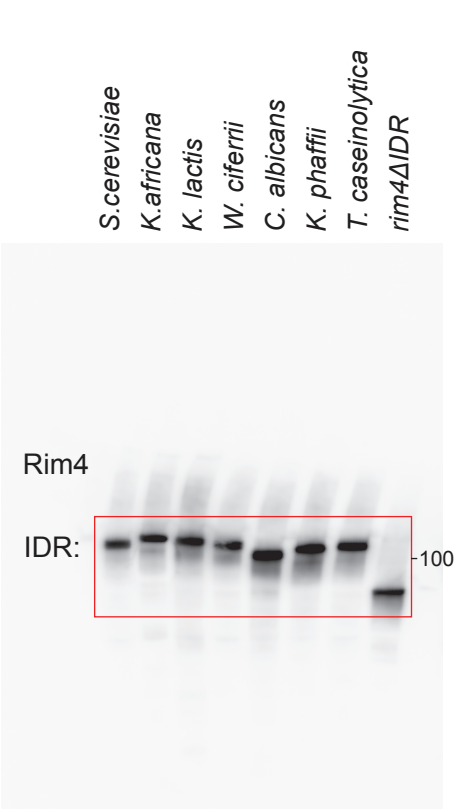

Pgk1 SDS-PAGE

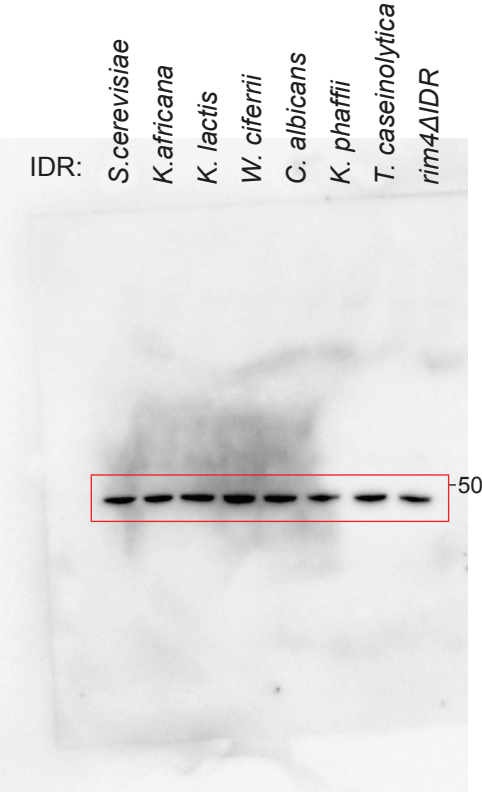

Supplement: S2 Data — (ZIP) [file pbio.3003396.s013.zip › S1_Raw_Images/Raw_Images_Fig4.pdf]

Rim4 SDS-PAGE

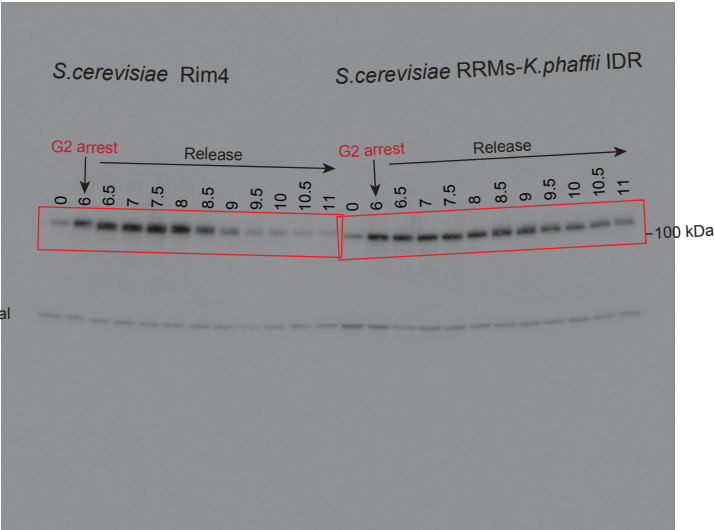

Cib3 SDS-PAGE

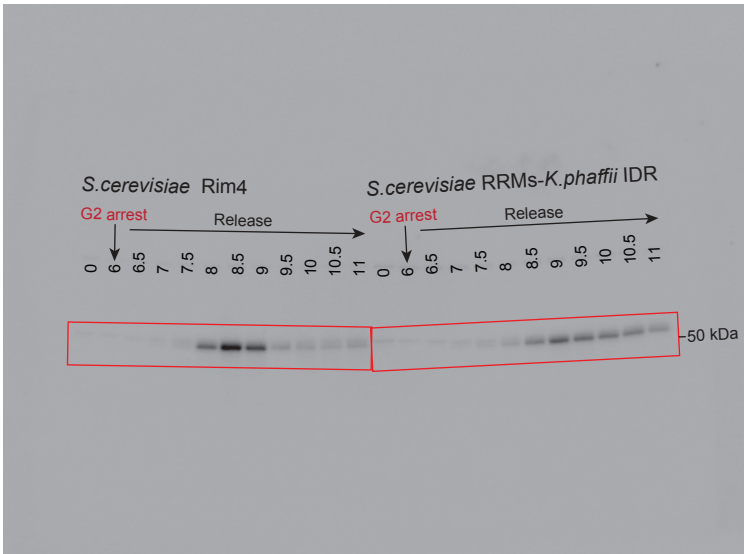

Pgk1 SDS-PAGE

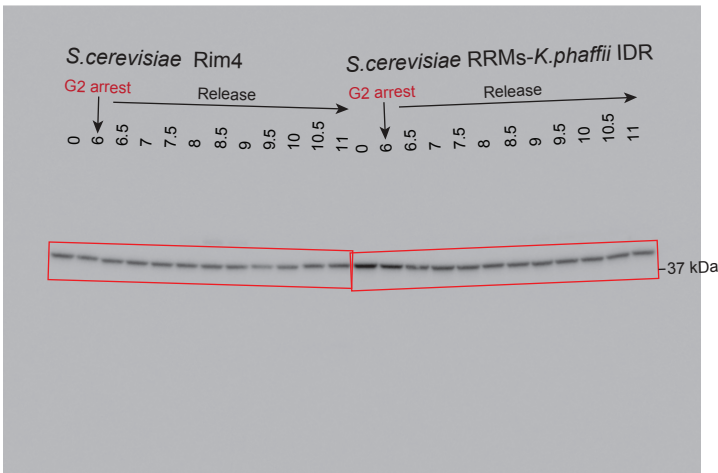

CLB3 Northern blot

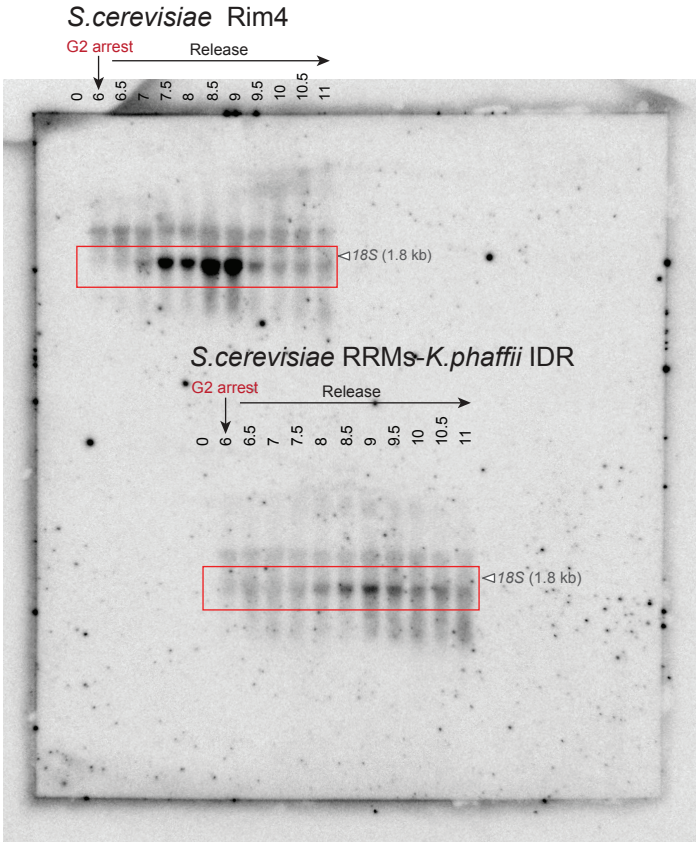

rRNA loading control

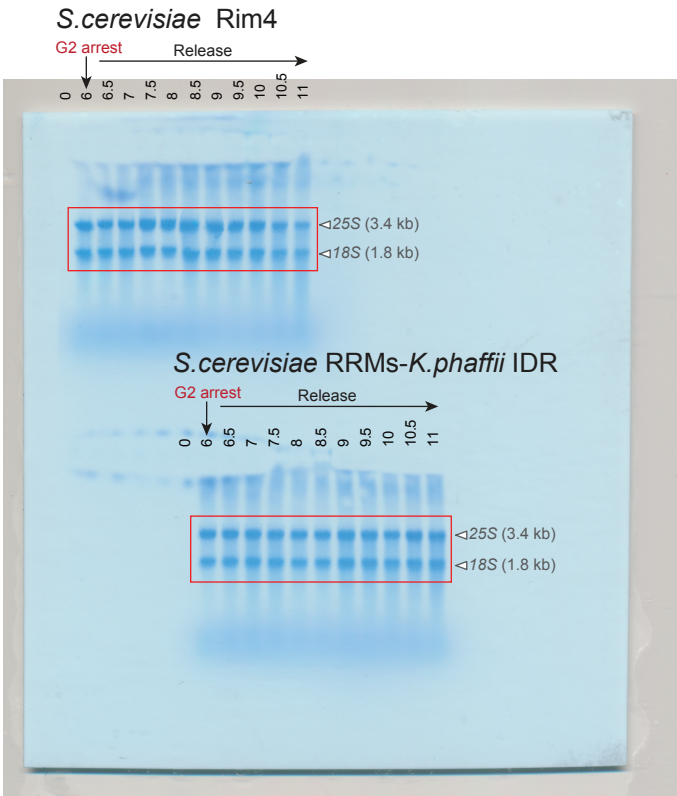

Supplement: S2 Data — (ZIP) [file pbio.3003396.s013.zip › S1_Raw_Images/Raw_Images_S6_Fig.pdf]

S7A

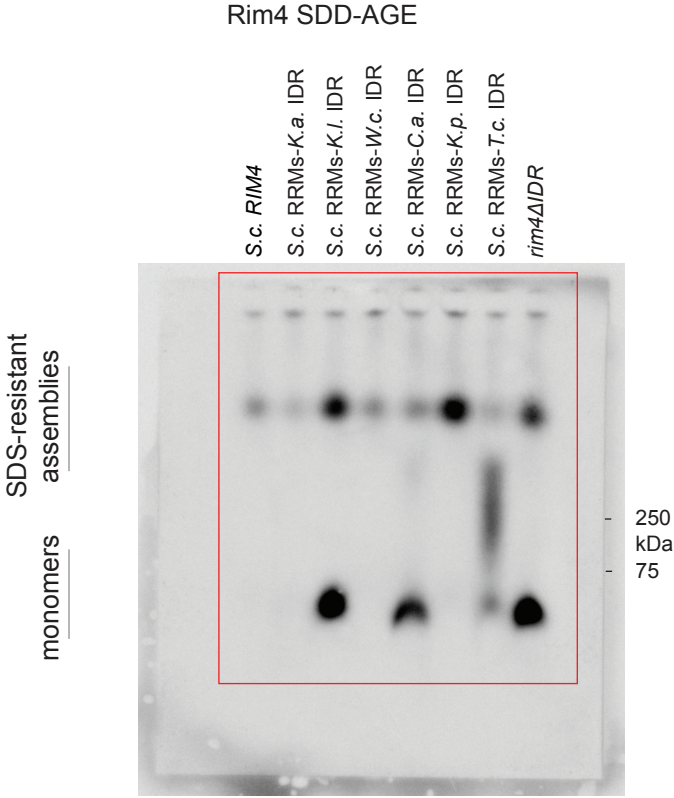

S7B

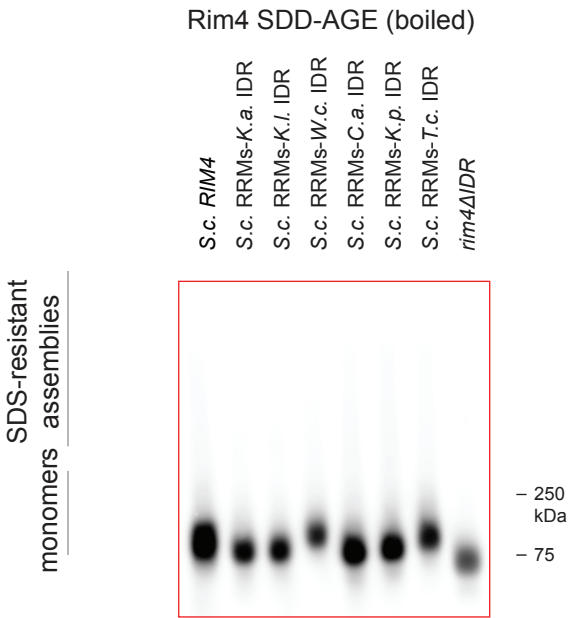

S7E

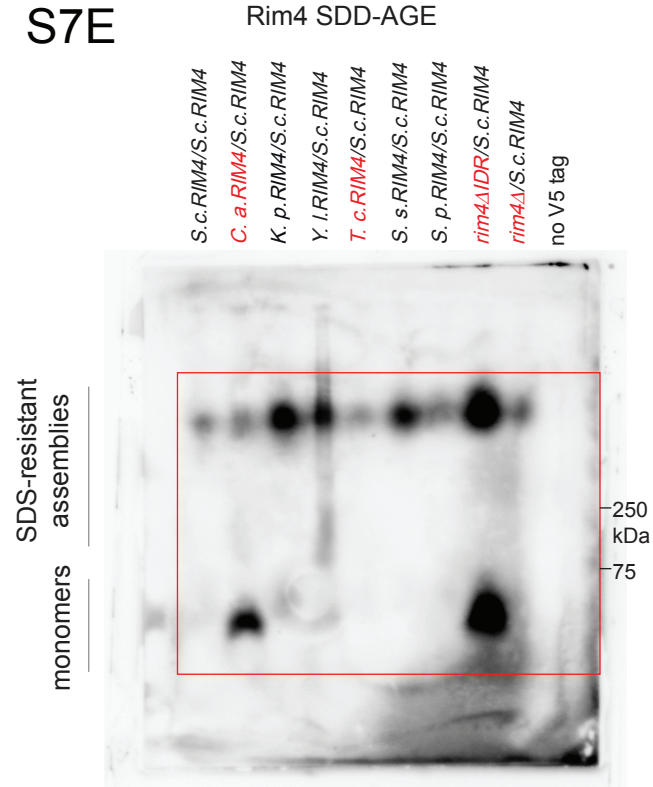

Rim4 SDS-PAGE

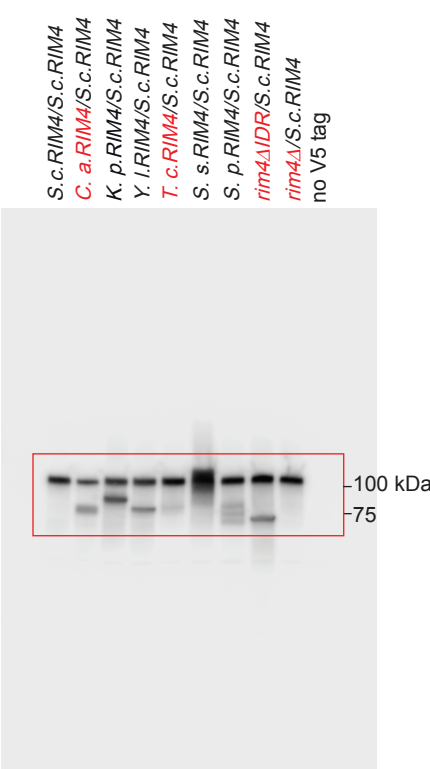

Pgk1 SDS-PAGE

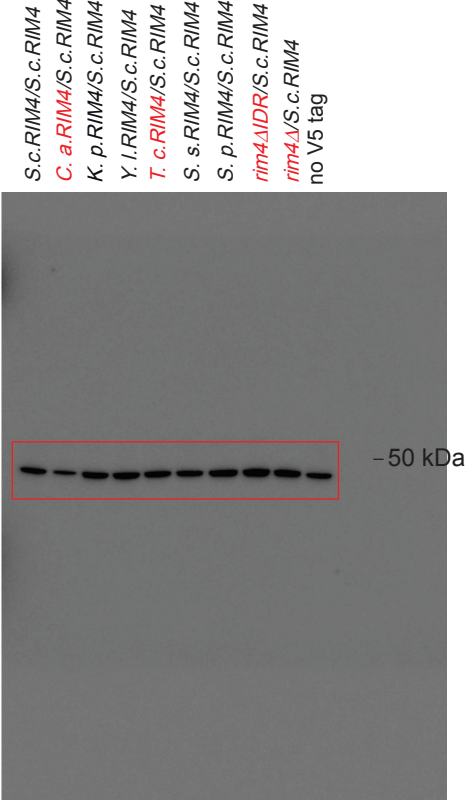

Supplement: S2 Data — (ZIP) [file pbio.3003396.s013.zip › S1_Raw_Images/Raw_Images_S7_Fig.pdf]

Rim4 SDS-PAGE

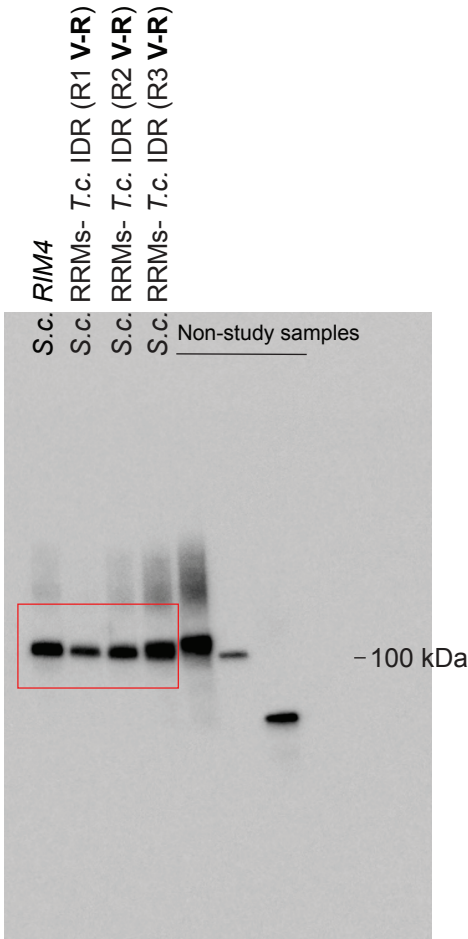

Pgk1 SDS-PAGE

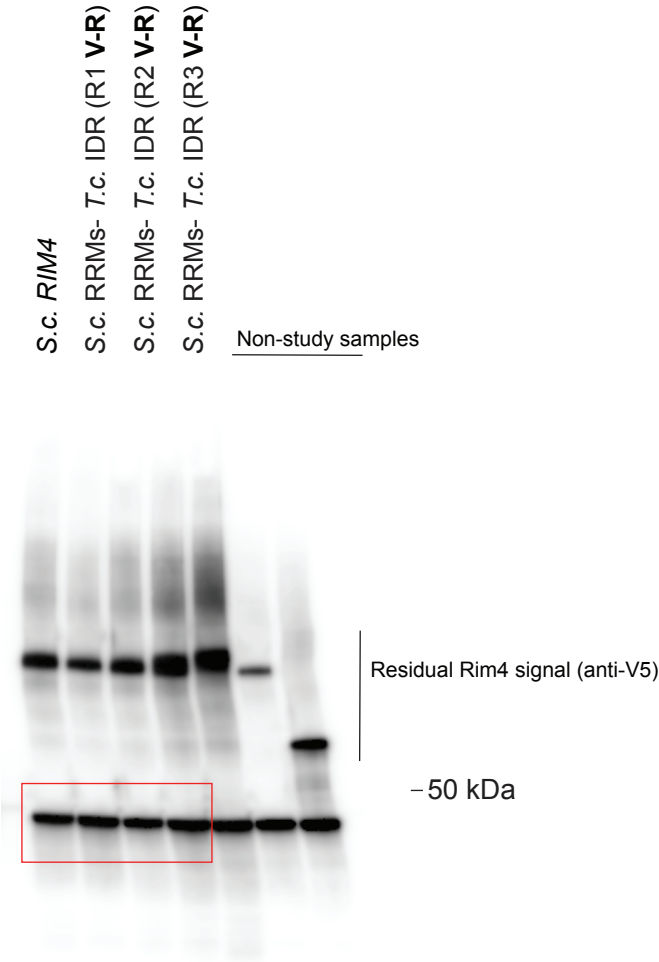

Supplement: S2 Data — (ZIP) [file pbio.3003396.s013.zip › S1_Raw_Images/Raw_Images_S9_Fig.pdf]
