# Supplementary material for: Cross‐species rescue reveals sequence requirements for a rapidly evolving intrinsically disordered region
Source: PLoS Biol. 2025 Sep 25;23(9):e3003396. doi: 10.1371/journal.pbio.3003396 (PMC12483275; doi:10.1371/journal.pbio.3003396)
Supplement: S2 Data — (ZIP) [file pbio.3003396.s013.zip › S1_Raw_Images/Raw_Images_Fig6.pdf]

6C

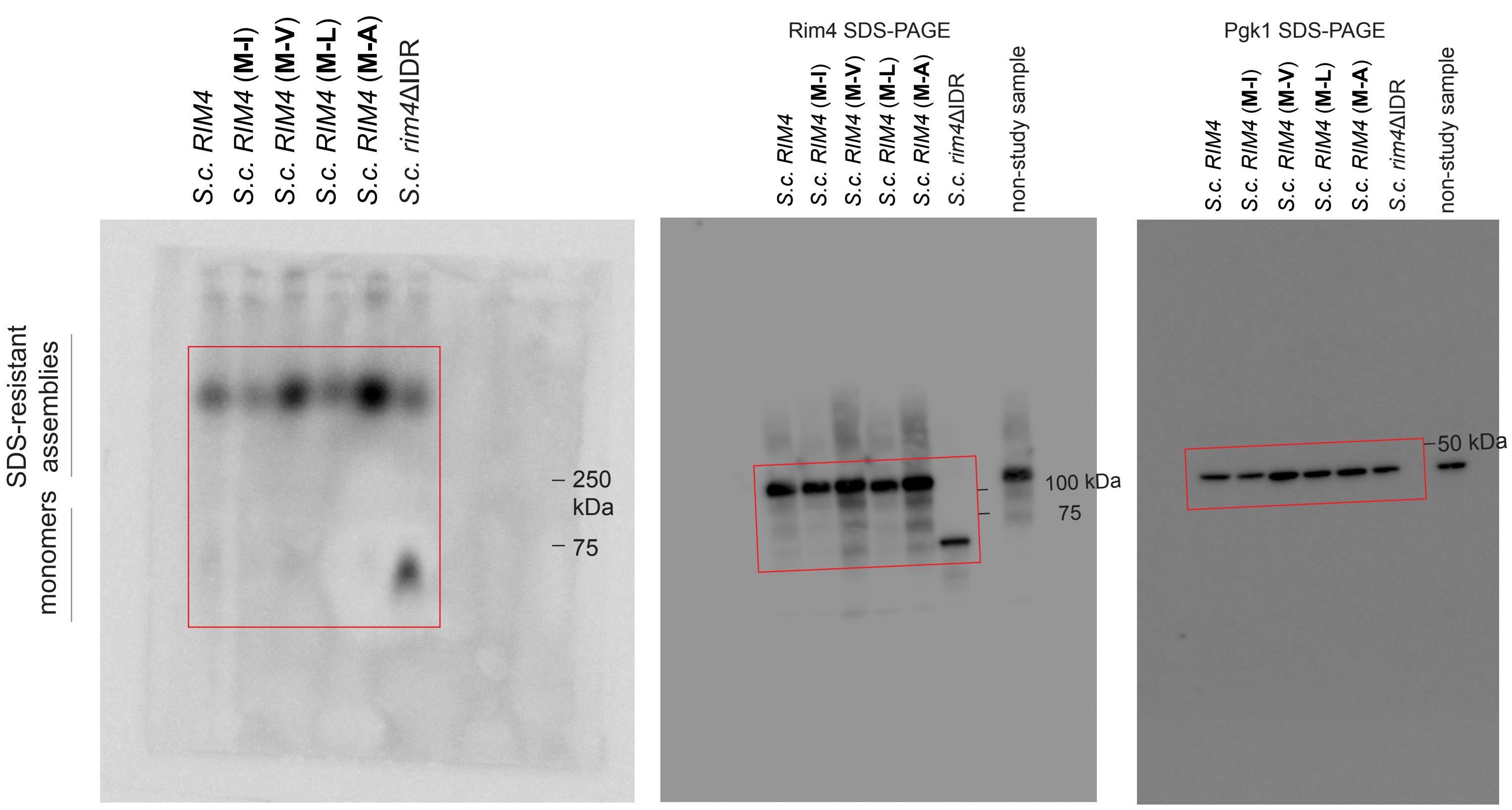
Rim4 SDD-AGE
S.c. RIM4
S.c. RIM4 (M-I)
S.c. RIM4 (M-V)
S.c. RIM4 (M-L)
S.c. RIM4 (M-A)
S.c. rim4ΔIDR
SDS-resistant assemblies
monomers
250 kDa
75
Rim4 SDS-PAGE
S.c. RIM4
S.c. RIM4 (M-I)
S.c. RIM4 (M-V)
S.c. RIM4 (M-L)
S.c. RIM4 (M-A)
S.c. rim4ΔIDR
non-study sample
100 kDa
75
Pgk1 SDS-PAGE
S.c. RIM4
S.c. RIM4 (M-I)
S.c. RIM4 (M-V)
S.c. RIM4 (M-L)
S.c. RIM4 (M-A)
S.c. rim4ΔIDR
non-study sample
50 kDa

6F

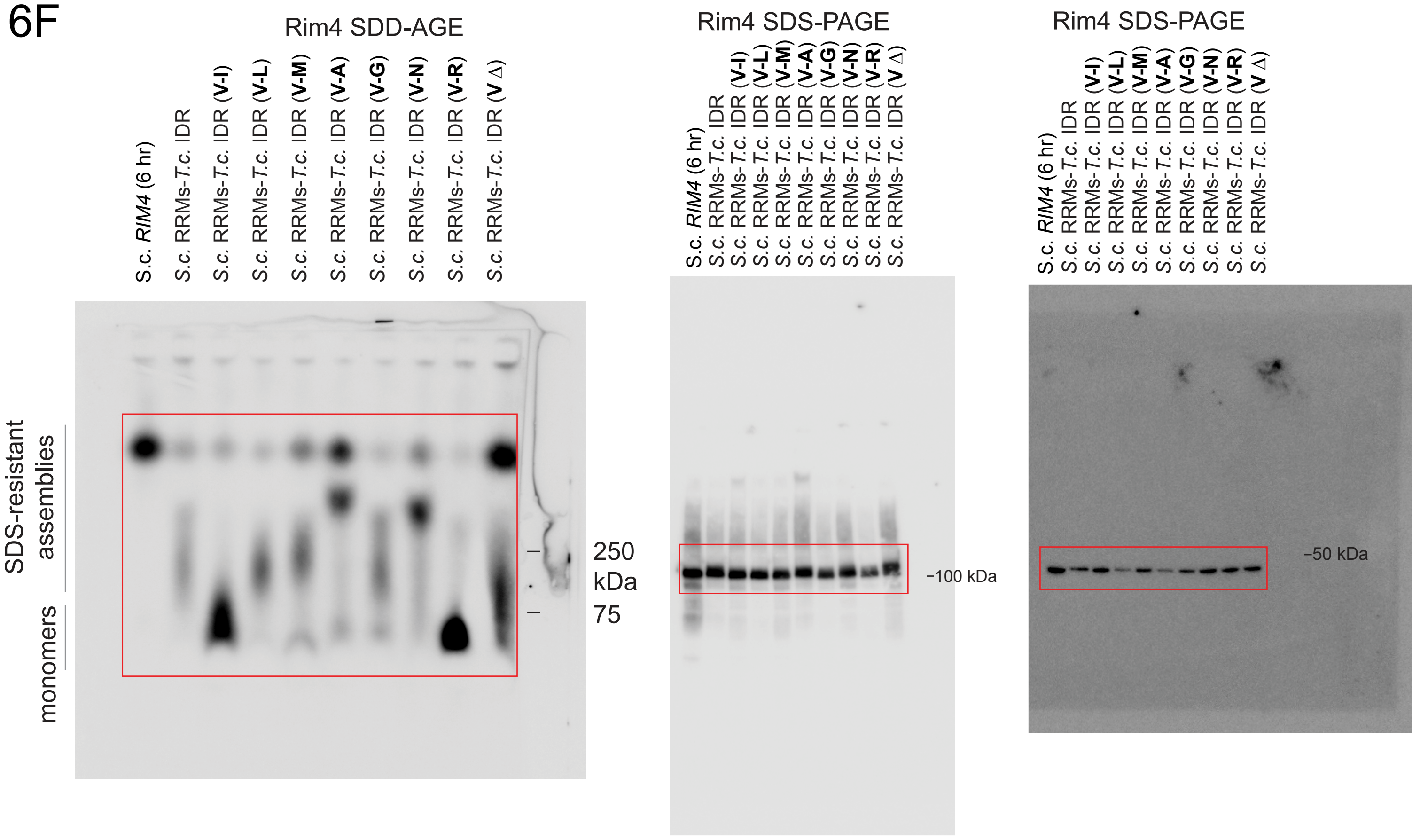
Rim4 SDD-AGE
S.c. RIM4 (6 hr)
S.c. RRMs-T.c. IDR
S.c. RRMs-T.c. IDR (V-I)
S.c. RRMs-T.c. IDR (V-L)
S.c. RRMs-T.c. IDR (V-M)
S.c. RRMs-T.c. IDR (V-A)
S.c. RRMs-T.c. IDR (V-G)
S.c. RRMs-T.c. IDR (V-N)
S.c. RRMs-T.c. IDR (V-R)
S.c. RRMs-T.c. IDR (V Δ)
SDS-resistant assemblies
monomers
250 kDa
75
Rim4 SDS-PAGE
S.c. RIM4 (6 hr)
S.c. RRMs-T.c. IDR
S.c. RRMs-T.c. IDR (V-I)
S.c. RRMs-T.c. IDR (V-L)
S.c. RRMs-T.c. IDR (V-M)
S.c. RRMs-T.c. IDR (V-A)
S.c. RRMs-T.c. IDR (V-G)
S.c. RRMs-T.c. IDR (V-N)
S.c. RRMs-T.c. IDR (V-R)
S.c. RRMs-T.c. IDR (V Δ)
100 kDa
Rim4 SDS-PAGE
S.c. RIM4 (6 hr)
S.c. RRMs-T.c. IDR
S.c. RRMs-T.c. IDR (V-I)
S.c. RRMs-T.c. IDR (V-L)
S.c. RRMs-T.c. IDR (V-M)
S.c. RRMs-T.c. IDR (V-A)
S.c. RRMs-T.c. IDR (V-G)
S.c. RRMs-T.c. IDR (V-N)
S.c. RRMs-T.c. IDR (V-R)
S.c. RRMs-T.c. IDR (V Δ)
50 kDa
