# Supplementary material for: Cross‐species rescue reveals sequence requirements for a rapidly evolving intrinsically disordered region
Source: PLoS Biol. 2025 Sep 25;23(9):e3003396. doi: 10.1371/journal.pbio.3003396 (PMC12483275; doi:10.1371/journal.pbio.3003396)
Supplement: S2 Data — (ZIP) [file pbio.3003396.s013.zip › S1_Raw_Images/Raw_Images_S1_Fig.pdf]

S1A

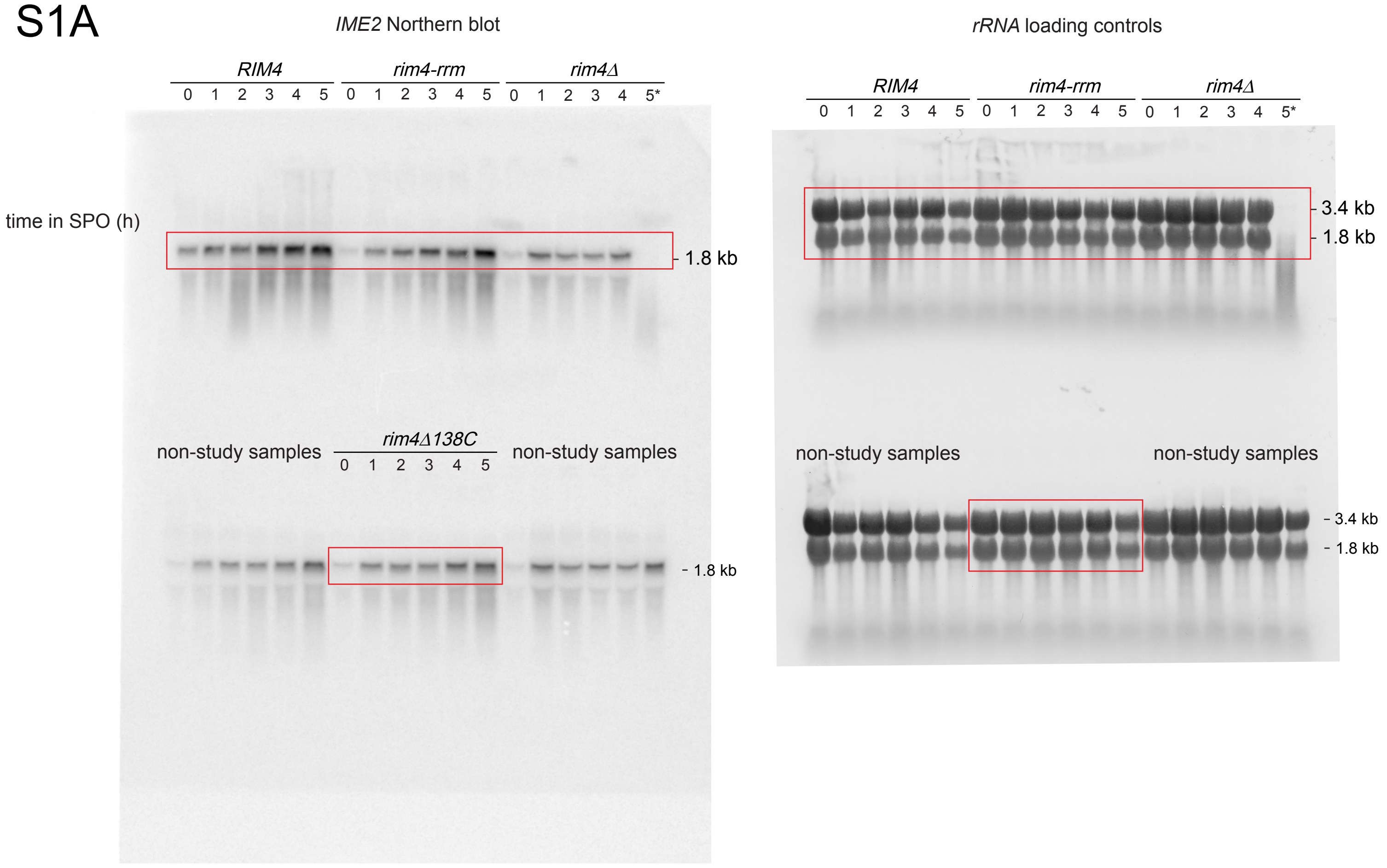
IME2 Northern blot
RIM4
rim4-rrm
rim4Δ
0 1 2 3 4 5 0 1 2 3 4 5 0 1 2 3 4 5*
time in SPO (h)
1.8 kb
non-study samples
rim4Δ138C
0 1 2 3 4 5
non-study samples
1.8 kb
rRNA loading controls
RIM4
rim4-rrm
rim4Δ
0 1 2 3 4 5 0 1 2 3 4 5 0 1 2 3 4 5*
3.4 kb
1.8 kb
non-study samples
non-study samples
3.4 kb
1.8 kb

S1B

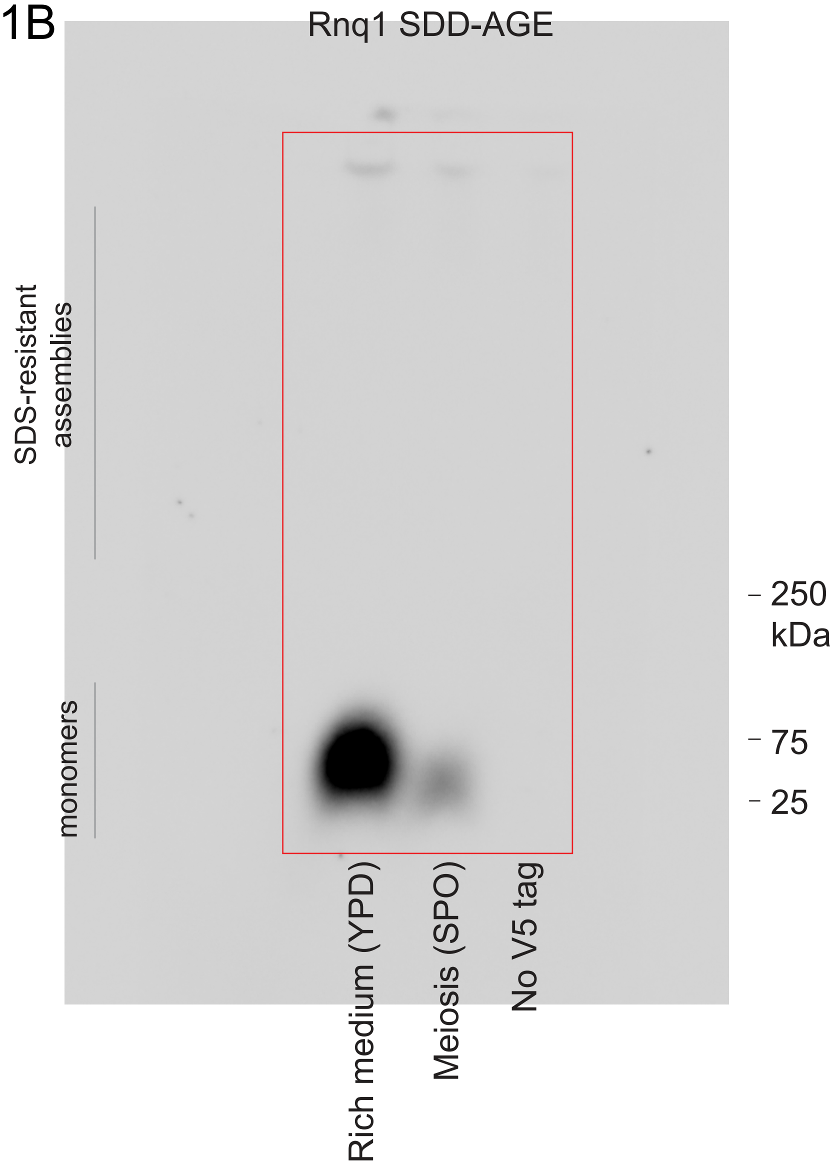
Rnq1 SDD-AGE
SDS-resistant assemblies
monomers
250 kDa
75
25
Rich medium (YPD)
Meiosis (SPO)
No V5 tag
